# COVID-19 outbreaks in residential aged care facilities: an agent-based modeling study

**DOI:** 10.3389/fpubh.2024.1344916

**Published:** 2024-05-21

**Authors:** Fenella McAndrew, Rachel Sacks-Davis, Romesh G. Abeysuriya, Dominic Delport, Daniel West, Indra Parta, Suman Majumdar, Margaret Hellard, Nick Scott

**Affiliations:** ^1^Burnet Institute, Melbourne, VIC, Australia; ^2^Department of Epidemiology and Preventive Medicine, Monash University, Melbourne, VIC, Australia; ^3^School of Population and Global Health, The University of Melbourne, Parkville, VIC, Australia; ^4^Victorian Government Department of Health, Melbourne, VIC, Australia; ^5^Department of Infectious Diseases, The Alfred and Monash University, Melbourne, VIC, Australia; ^6^Department of Infectious Diseases, The University of Melbourne and Victorian Infectious Diseases Reference Laboratory, Parkville, VIC, Australia

**Keywords:** COVID-19, agent-based model, outbreak, residential aged care facility, vaccination, non-pharmaceutical interventions

## Abstract

**Introduction:**

A disproportionate number of COVID-19 deaths occur in Residential Aged Care Facilities (RACFs), where better evidence is needed to target COVID-19 interventions to prevent mortality. This study used an agent-based model to assess the role of community prevalence, vaccination strategies, and non-pharmaceutical interventions (NPIs) on COVID-19 outcomes in RACFs in Victoria, Australia.

**Methods:**

The model simulated outbreaks in RACFs over time, and was calibrated to distributions for outbreak size, outbreak duration, and case fatality rate in Victorian RACFs over 2022. The number of incursions to RACFs per day were estimated to fit total deaths and diagnoses over time and community prevalence.

Total infections, diagnoses, and deaths in RACFs were estimated over July 2023–June 2024 under scenarios of different: community epidemic wave assumptions (magnitude and frequency); RACF vaccination strategies (6-monthly, 12-monthly, no further vaccines); additional non-pharmaceutical interventions (10, 25, 50% efficacy); and reduction in incursions (30% or 60%).

**Results:**

Total RACF outcomes were proportional to cumulative community infections and incursion rates, suggesting potential for strategic visitation/staff policies or community-based interventions to reduce deaths. Recency of vaccination when epidemic waves occurred was critical; compared with 6-monthly boosters, 12-monthly boosters had approximately 1.2 times more deaths and no further boosters had approximately 1.6 times more deaths over July 2023–June 2024. Additional NPIs, even with only 10–25% efficacy, could lead to a 13–31% reduction in deaths in RACFs.

**Conclusion:**

Future community epidemic wave patterns are unknown but will be major drivers of outcomes in RACFs. Maintaining high coverage of recent vaccination, minimizing incursions, and increasing NPIs can have a major impact on cumulative infections and deaths.

## 1 Introduction

COVID-19 has been an established global threat since its emergence in 2019. As of May 2023, there have been over 760 million diagnosed cases, 7 million deaths and an estimated 27 million cumulative excess deaths reported globally (1–3). In 2022 COVID-19 was listed as Australia’s third leading cause of death after ischaemic heart disease and dementia (4). Residential aged care facilities and other long-term care facilities (RACFs) have experienced a disproportionate number of deaths from COVID-19 (5); for example, in 2020, 75% of COVID-19 related deaths in Australia occurred in RACFs (6). Even since vaccines and treatments have become available, a high burden in RACFs persists; between January 2022 and April 2023, 26% of Australian COVID-19 related deaths occurred in RACFs, with COVID-19 recorded as the cause of 5.4% of all resident deaths (7).

Australia has implemented multi-layered COVID-19 measures within RACFs to reduce infections, hospitalisations, and deaths. These have included testing (symptomatic and surveillance), screening of residents during outbreaks, isolation of positive cases, quarantine of close contacts, masks, face shields, and during waves of infection in 2020–2021, visitation limits and complete facility lockdowns (8, 9). Additionally, RACFs were given priority vaccine allocations for staff and residents in 2021, and antivirals for residents in 2022 (10, 11). While the interventions and policies in place have changed over time, RACFs have typically employed more interventions and greater levels of restrictions than the general community to protect vulnerable residents (12).

Over the first two years of the COVID-19 pandemic, the restrictions applied to RACFs are likely to have had important impacts preventing COVID-19 mortality, particularly in the absence of vaccination (13). However, when considering longer-term COVID-19 responses in RACFs there is a need to acknowledge the trade-off between levels of protections and quality of life for residents, particularly for restrictive interventions (14). This trade-off, in the current context of vaccinations and therapeutics to reduce morbidity and mortality, makes it critical to ensure that any implemented interventions are evidence-based and effective. Quantifying the potential impact of interventions can help facilitate informed and objective discussions on how best to balance COVID-19 risk with quality of life.

Modeling can be used to assess and compare the effectiveness of different interventions for COVID-19 in RACFs. Agent-based models are most suited for simulating smaller cohorts, such as in RACFs, due to the ability to specify detailed contact networks (15, 16), and have been used to assess the effectiveness of personal protective equipment (PPE), testing, isolation, vaccination strategies, and immunity (17–19). Other types of models, including equation-based models, have also been used to assess the impact of ventilation systems on infection rates (20). While existing models have considered the outcomes of outbreaks, to our knowledge they have not considered how community prevalence influences rates of incursions, and hence the relationships between community epidemic waves, incursions, RACF interventions, and health outcomes.

The objective of this analysis was to investigate the relationship between community COVID-19 prevalence; incursion rates into RACFs; and subsequent outcomes of infections, diagnoses, and deaths when different interventions are in place. Using an agent-based model to quantify the effectiveness of different interventions for minimizing COVID-19 deaths, RACF outcomes were projected under different community prevalence and RACF intervention scenarios. These outcomes can inform discussions on the trade-off between interventions that reduce COVID-19 risk but impact on quality of life for residents, and strategies for triggering different testing, vaccination, and non-pharmaceutical interventions (NPIs) to mitigate the impacts of future epidemic waves.

## 2 Methods

### 2.1 Modeling methodological overview

This study was done in two parts. First, an agent-based model was parametrized and calibrated to allow simulation of realistic *individual* outbreaks in RACFs under different scenarios, such that after multiple stochastic simulations the distribution of diagnoses and deaths per outbreak matched data from outbreaks in the state of Victoria. Second, to model expected total annual state-wide RACF COVID-19 outcomes (infections, diagnoses and deaths), the rate of incursions into RACF was estimated, accounting for different plausible community prevalence profiles, to inform how many individual outbreak simulations should be performed in each month.

The structure of the paper is as follows. Sections 2.2, 2.3 and 2.4 outline Victorian RACF populations and how they are approximated in the model. Sections 2.5 and 2.6 describe the approach taken to simulate individual outbreaks, and to use these to estimate total state-wide outcomes, respectively. Sections 2.7 and 2.8 describe how the interventions were modeled, including immunity from vaccination and post-exposure. Sections 2.9–2.11 describe the model calibration to produce realistic individual outbreaks (2.9), how these were converted to probability distributions for further sampling based on estimates of the number of outbreaks per month (2.10; “incursion-outcome libraries”), and model calibration to state-wide outcomes (2.11). Section 2.12 describes the intervention/policy scenarios and sensitivity analyses undertaken.

### 2.2 Setting

The model considers RACFs in Victoria, Australia’s second most populous state. The Australian Institute of Health and Welfare reported in 2023 that Victoria has 754 RACFs, which vary in size (median 58 residents [inter-quartile range 10–150]; median 82 staff [inter-quartile range 11–198]). Of the 754 RACFs, 34.8% are privately operated, 8.5% are government operated and 56.7% are not for profit operated (21); all are considered together for this analysis (see limitations).

### 2.3 Data

The model was informed by data collected from the Victorian Department of Health on outbreaks that occurred in Victorian RACFs between May 2020 and February 2023 (22). For each outbreak, the dataset included: number of diagnoses and deaths for residents and staff, date of first and last diagnosis associated with the outbreak, number of occupying residents at the time of the outbreak, and whether it was a private or public facility. The calibration process only requires data between February 2022 and February 2023, so data before this period is not used for this study (Appendix B).

Additional demographic data was available for all Victorian RACFs (number of staff and residents per RACF) and at a state-level (aggregated vaccine and treatment coverage over time) to inform the model population (see Appendix A.1).

### 2.4 Model overview

An established agent-based model, *Covasim,* was used to simulate outbreaks in RACFs (23). The model was developed by the Burnet Institute and the Gates Foundation and is available online (24). Covasim has previously been used to model epidemic waves and response strategies in Australia (25–27), as well as outbreaks in schools (28). *Covasim* enables specification of population size and age structure, as well as transmission networks between different agents. *Covasim* calculates the transmission probability between agents for each time step, which can change over the simulation due to agents’ immunity from previous vaccination or infection and interventions in place. Agents can be assigned individual probabilities from moving from one state to another (susceptible to infected, or critically ill to dead) based on their assigned age.

**Figure 1:**
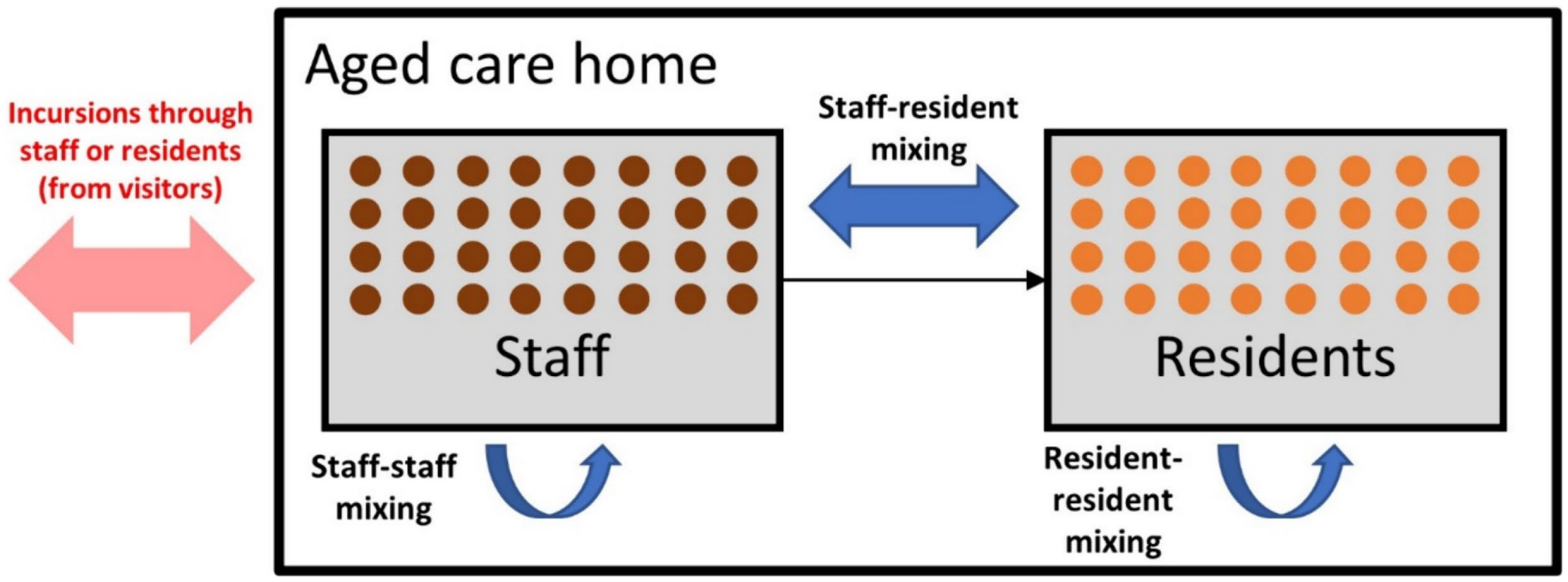
Model schematic displaying the incursion source and the three types of contacts within residential aged care facilities (RACFs).

Within an RACF in the model, two types of agents were defined (residents and staff) with three types of interactions between them (resident-resident, staff-staff, and staff-resident) (Figure 1). Visitors into RACFs were not explicitly modeled, but could be interpreted as the source of an incursion, seeding an infection in either a staff member or resident. Staff were randomly selected from the general working-age population (aged between 18 and 65) and residents were randomly selected from the older adult population (aged 80 years and over).

Each time a simulation was run the RACF size, resident age distribution, and staff-to-resident ratio were sampled from their empirical distributions (Appendix A1).

### 2.5 Individual outbreak simulations

The model runs by generating a single RACF at random (i.e., sampling RACF characteristics), and simulating a single incursion by infecting either a resident or a staff member. The COVID-19 variant used to infect the staff member or resident was randomly sampled from the distribution of variant prevalence in Victoria on the simulation date; the Omicron variants modeled include BA.2, BA.4/BA.5 and XBF (Appendix A6). Following an incursion, transmission can occur between contacts, and either symptomatic testing or surveillance testing is required to detect the first case. Once a case is identified all resident contacts are tested daily with rapid antigen tests (RATs), and all positive cases are assumed to isolate. Upon initial diagnosis, the facility is assumed to adopt some form of NPIs for risk mitigation that reduces transmission risk by 66% (calibrated to fit outbreak duration data).

As the model is stochastic, different outbreak sizes can occur within each simulation, including no cases being detected. This may happen because transmission does not occur following an incursion, an infected staff or resident may not be symptomatic (depending on age, vaccine history, and exposure-acquired immunity), symptomatic individuals may not test, and RATs may return false negative results. In addition, infected people are not necessarily detected if testing occurs during their incubation phase.

Each simulation is run for 3 months post-incursion to ensure sufficient time for the outbreak to conclude. At the end of each individual outbreak simulation, the total number of infections, diagnoses, and COVID-19 deaths for residents and staff, as well as the duration of the outbreak, are recorded as model outcomes for further analysis and comparison.

### 2.6 Estimating state-wide outcomes

Each month many incursions into RACFs occur across Victoria, and hence multiple outbreak simulations must be run to reproduce expected state-wide total infection, diagnoses and COVID-19 deaths in RACFs. For each month between July 2023 and June 2024 inclusive, many individual stochastic outbreaks are simulated, so that state-wide total infections, diagnoses and COVID-19 deaths can be estimated as the aggregate of multiple outbreaks per month (the number varies by month and is determined through calibration). For this analysis, where an individual outbreak spans multiple months, infections, diagnoses and COVID-19 deaths are assigned to the month it was first detected in.

### 2.7 Interventions

The model includes options for NPIs (e.g., interventions to improve air quality or mask wearing, which reduce transmission risks per contact), symptomatic testing (polymerase chain reaction [PCR] tests or RATs), routine surveillance testing (staff and/or residents), outbreak management testing (one-off test of entire RACF or daily testing of contacts), and vaccination (next section) (29).

The baseline scenario assumes no surveillance testing and an outbreak management testing algorithm of daily testing of resident close contacts with RATs for 7-days after the positive case is identified (29, 30). The symptomatic testing probability for residents and staff is 70 and 50% per week, respectively, and both residents and staff have a 3% per week probability for asymptomatic testing (i.e., being asymptomatic for COVID-19 but symptomatic for another illness).

### 2.8 Population immunity

To accurately capture population immunity at the time of the incursion, we accounted for historical vaccination, exposure-acquired immunity, and waning immunity. For vaccination, an initial two-dose coverage of 98% for residents and 100% for staff was modeled and assumed to be reached by October 2021 (10, 31). Following this, time-varying vaccine booster coverage was used to capture increasing and waning immunity within RACFs (Appendix A.4). From July 2023–June 2024 the model assumes rolling 6-monthly vaccine doses for RACF residents, reaching a coverage of 80%.

Because we are only simulating a single outbreak in this study, we do not explicitly model previous infections. However, exposure-acquired immunity from past infections is important for setting initial conditions in the RACF before the simulated incursion occurs. Historical data for the weekly proportion of aged care residents diagnosed was used to approximate exposure-acquired immunity in RACFs over time; for each week, an equivalent proportion of residents in the model were assigned immunity equivalent to an infection occurring in that week. Residents with lower levels of base immunity were prioritized over those with higher immunity to approximate their increased susceptibility. A similar process was used for staff, except based on community prevalence estimates rather than resident diagnoses. Due to uncertainty in future epidemic waves, a simple function was used for community prevalence from February 2023 onwards; in the baseline a new epidemic wave was assumed to occur every 5 months, with magnitude approximately equal to the 2022 August and December waves.

### 2.9 Model calibration: outcomes per incursion

The model was calibrated to Victorian RACF outbreak data in December 2022; specifically, the distribution of outbreak size (among patients and staff), the distribution of outbreak duration, and case fatality rate among residents (total deaths / total diagnosed) (32). All calibration was done manually and enabled the model to be constrained so that simulation outcomes were more realistic and aligned with Victorian RACF data. Calibration to outbreak size and duration was achieved by adjusting the mean number of contacts per resident and staff per day, and the relative risk of transmission between contacts (staff-staff, resident-resident, staff-resident). Calibration to case fatality rate was achieved by adjusting the probability of death given infection among residents.

### 2.10 Incursion-outcome libraries

Once the model was calibrated to outcomes per incursion, such that the distribution of stochastic outcomes after simulating many individual outbreaks aligned with the observed data, the model could be used to generate an “incursion-outcome library” that specifies the probability of different outcomes following an incursion. Since outbreak risks change over time due to variants in circulation, and waning immunity, as well as for any policy combinations, an incursion-outcome library was created for each calendar month and each policy scenario based on the results of 1,000 simulated incursions (a large enough number that the distributions did not change when further simulations were run). The incursion-outcome library for each month and policy scenario can be repeatedly sampled to approximate outcomes following multiple independent incursions; for example aggregate outcomes from N samples are an estimate of plausible RACF outcomes after N incursions, and bootstrapping is used to estimate expected values and uncertainty intervals following N incursions.

### 2.11 Model calibration: state-wide outcomes per month

Data was available on total state-wide diagnoses and deaths in RACFs per calendar month from February to December 2022, which are the result of many incursions taking place each month. The model produces a distribution of outcomes per incursion as described above (the incursion-outcome libraries for each month), but to reproduce the total number of RACF infections, diagnoses and deaths an estimate of the number of incursions per month is required. Multiple functional forms were tested to parametrize the number of incursions into RACFs over time, including constants, step functions timed with the emergence of different sub-variants, and rates proportional to community infections (Appendix B). Each choice of functional form gave a number of incursions per month, for which the corresponding incursion-outcome library was sampled to produce an estimate of state-wide total infections, diagnoses and COVID-19 deaths (with bootstrapping to obtain confidence intervals for monthly totals).

### 2.12 Scenarios and sensitivity analyses

For each scenario, the number of incursions per month were estimated (based on the functional form section 2.10), and total infections, diagnoses, and deaths were obtained by randomly sampling with replacement from the corresponding month and policy-specific incursion-outcome library. Bootstrapping was used to generate median and 95% uncertainty intervals for outcomes in each scenario and month.

Total infections, diagnoses and COVID-19 deaths for each month July 2023–June 2024 were added to give 12-month outcomes, with month-by-month results presented in the Appendix.

The following scenarios were considered based on plausible values and policies identified in collaboration with the Victorian Department of Health:

1. **Outbreak management testing**: resident contacts RAT tested daily (baseline), not tested, whole RACF tested once upon initial diagnosis.
2. **Symptomatic testing probability:** baseline (70%), very low (5%), low (50%), high (90%).
3. **Routine surveillance testing**: none (baseline), resident only, staff only, both staff and residents.
4. **Future epidemic wave assumptions**: community epidemic waves every 5-months (baseline), 7-months, 5-months with decreasing peaks, 5-months with decreasing peaks and increasing troughs.
5. **Additional NPIs**: with efficacy 0% (no additional NPIs, baseline), 10, 25, 50%.
6. **Vaccine rollout**: 6-monthly rolling boosters (baseline), 12-monthly rolling boosters, no additional boosters after 6th dose.

Three sensitivity analyses were performed:

1. **RACF incursions per community infections**: 3 per 1,000 (baseline), 1 per 1,000, 2 per 1,000, 4 per 1,000, 5 per 1,000.
2. **Incursions target**: Random (baseline), resident, staff.
3. **Number of incursions**: 1 (baseline), 2, 3.

The type of NPIs were not specified due to uncertainty in individual NPI efficacy and variation between RACFs according to baseline conditions and quality of implementation. However the NPI scenarios can refer to any interventions that could reduce transmission risks such as social distancing, mask wearing, portable HEPA filters or germicidal ultraviolet light (GUV) devices (33, 34). NPIs were applied to both staff and residents with the same assumed efficacy.

The future epidemic wave scenarios were chosen to investigate how the future prevalence of COVID-19 in the community could impact RACFs. The frequency and magnitude of the epidemic waves in these scenarios are not intended to be forecasts.

Only residents were targeted in the vaccine scenarios. Coverage of ongoing vaccine boosters was assumed to be imperfect, reaching 80% coverage of the resident population per vaccine round, with only residents who had received all previous boosters considered eligible for the additional dose.

## 3 Results

### 3.1 Baseline

**Figure 2:**
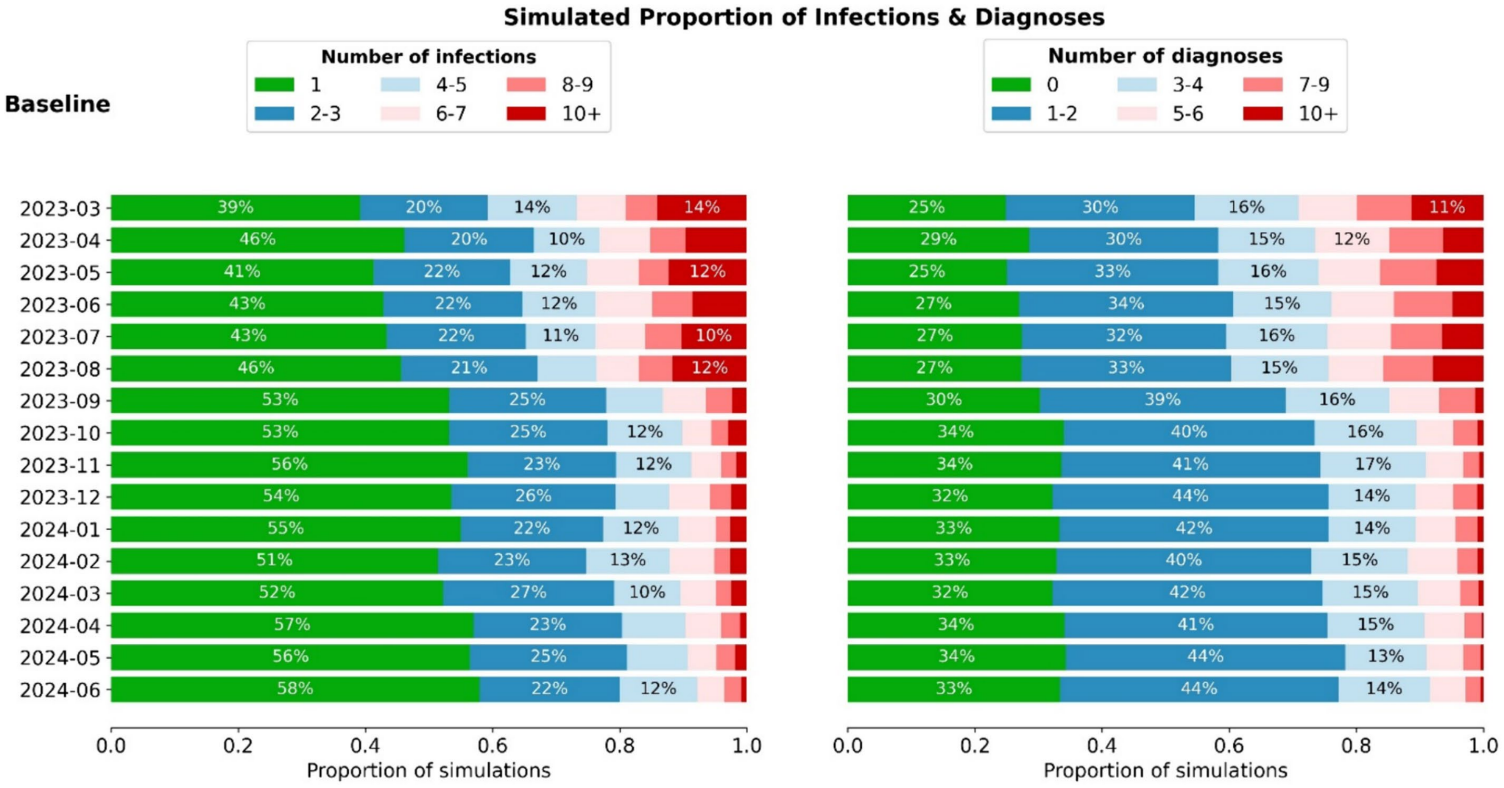
Distribution of infection and diagnoses outcomes (aggregate among staff + residents) for incursions occurring in each month between March 2023 and June 2024. When combined with the estimated number of incursions, these distributions can be used to estimate the total outcomes per month (Figure 3).

Figure 2 shows the baseline scenario incursion outcome libraries for each month between July 2023 and June 2024, assuming ongoing 5-monthly community epidemic waves, 6-monthly vaccine boosters for residents and no changes to testing or outbreak management. The probability of different outcomes following a single incursion into an RACF changes over time, with the risk of larger outbreaks reducing due to greater exposure-acquired immunity and vaccine boosters administered in August 2023 and February 2024. A decrease in outbreak size is observed from August to September 2023 attributed to the marked increase in resident immunity from a concurrent epidemic wave and round of booster doses (Figure 2). From September 2023 onwards booster rollout and epidemic waves are no longer aligned, and residents maintain a consistent level of immunity from both sources.

The number of incursions into RACFs each month was determined to be proportional to community prevalence, with a best fit of 3 RACF incursions per 1,000 community infections. Other relationships that were tested resulted in poorer model fits (Appendix B3).

**Figure 3:**
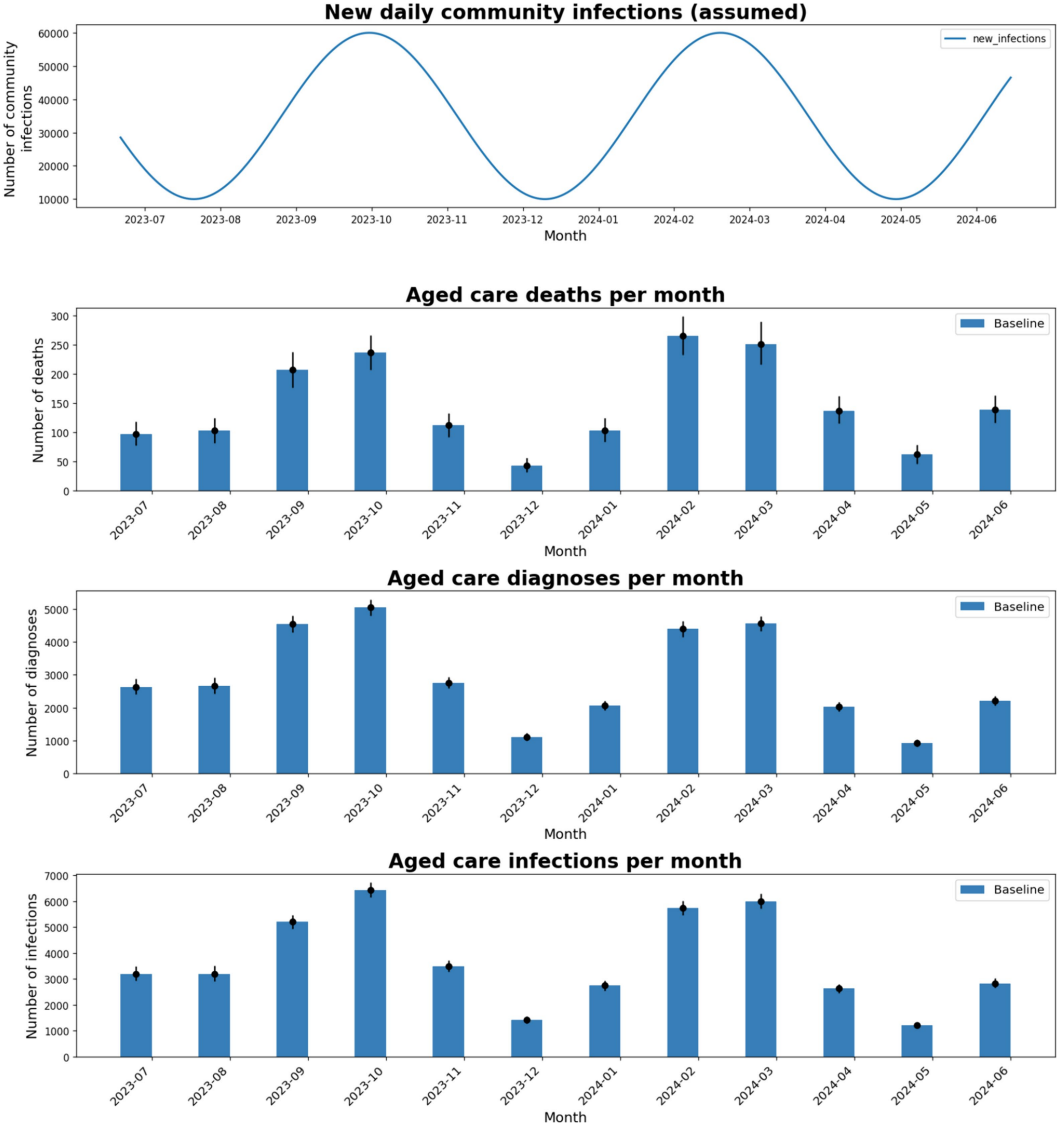
Total number of deaths, diagnoses, and infections in the model for each month between July-2023 and June-2024, using baseline intervention coverages and an assumed 5-monthly epidemic wave of the same magnitude as the Aug and Dec 2022 epidemic waves.

Total monthly infections, diagnoses, and deaths within Victorian RACFs in the model are obtained by sampling and aggregating incursion outcomes depending on the assumed community prevalence. Monthly outcomes in RACFs follow similar trends to the projected community prevalence (Figure 3). A slight decrease in peak RACF outcomes is observed for subsequent epidemic waves, due to greater exposure-acquired immunity and vaccine boosters administered.

### 3.2 Different assumptions for future community prevalence

**Figure 4:**
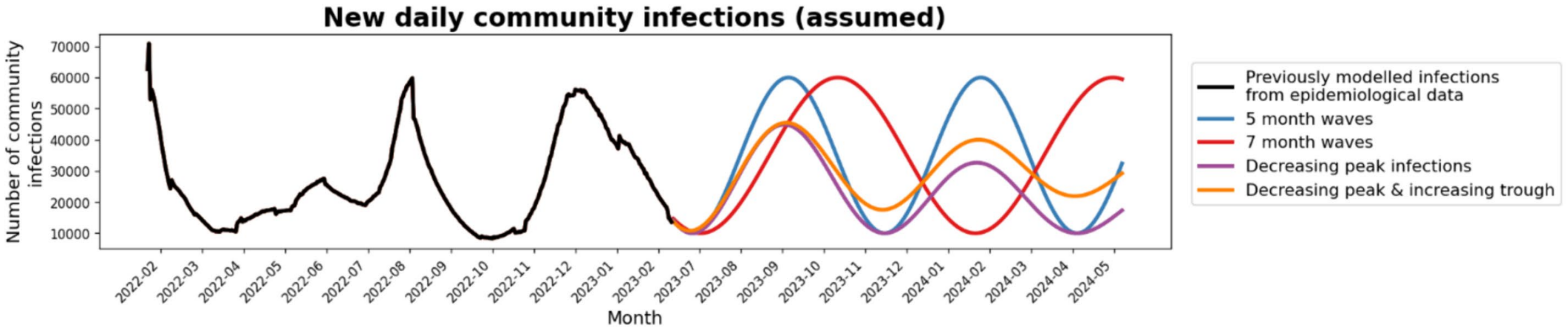
Community prevalence scenarios: Four future community prevalence scenarios were tested; 5-monthly waves (blue), 7-monthly waves (red), waves with decreasing peak infections (purple) and waves with decreasing peak infections and increasing troughs (orange).

Future community epidemic waves are unknown, so multiple different scenarios were modeled (Figure 4).

Total outcomes in RACFs were largely dependent on the area under the community infection curve for all community epidemic wave scenarios. Epidemic waves with similar peak heights had similar peak outcomes in RACFs (Appendix C) and waves with longer time between peaks has similar cumulative outcomes (Table 1). When future community epidemic waves were modeled to have lower peaks but higher troughs, cumulative outcomes were 17% less than the baseline (Table 1). Outcomes were best if community infections decreased over time, with a 44% reduction in cumulative outcomes.

**Table 1:** Percentage change in median cumulative infections, diagnoses and deaths for scenarios compared to the baseline.

Scenario		Difference compared to baseline scenario		
		Infections	Diagnoses	Deaths
Community infections	5-month waves	Baseline	Baseline	Baseline
	7-month waves	+3% (1, 5%)	+2% (0, 4%)	0% (−5, 4%)
	Decreasing peak infections	−44% (−45, −43%)	−44% (−46, −43%)	−44% (−48, −41%)
	Increasing trough infections	−10% (−12, −8%)	−10% (−12, −8%)	−17% (−21, −12%)
Outbreak management testing	Residents tested daily	Baseline	Baseline	Baseline
	No tracing	+6% (4, 8%)	−23% (−25, −22%)	+1% (−3, 6%)
	Home tested once off	−14% (−16, −13%)	−7% (−9, −5%)	−13% (−18, −9%)
Routine surveillance testing	No surveillance testing	Baseline	Baseline	Baseline
	Staff surveillance testing	−43% (−44, −41%)	−39% (−40, −37%)	−50% (−53, −47%)
	Resident surveillance testing	−27% (−28, −25%)	−7% (−9, −5%)	−32% (−35, −27%)
	Staff & resident surveillance testing	−53% (−54, −52%)	−39% (−40, −38%)	−61% (−64, −58%)
Symptomatic testing	Medium testing	Baseline	Baseline	Baseline
	High testing	−16% (−18, −15%)	−5% (−6, −3%)	−21% (−26, −17%)
	Low testing	+19% (17, 21%)	0% (−1, 2%)	+16% (11, 21%)
	Very low testing	+89% (86, 93%)	−22% (−24, −20%)	+94% (87, 101%)
NPI efficacy	NPI 0% efficacy	Baseline	Baseline	Baseline
	NPI 10% efficacy	−12% (−14, −11%)	−13% (−15, −12%)	−18% (−22, −14%)
	NPI 25% efficacy	−30% (−31, −29%)	−31% (−33, −30%)	−32% (−36, −28%)
	NPI 50% efficacy	−51% (−52, −50%)	−55% (−56, −54%)	−56% (−59, −53%)
Vaccine booster	6-month booster	Baseline	Baseline	Baseline
	12-month booster	+70% (67, 73%)	+77% (74, 81%)	+23% (18, 29%)
	No booster	+146% (142, 151%)	+163% (158, 168%)	+62% (55, 68%)

### 3.3 Testing interventions

The baseline scenario has no routine surveillance testing of staff or patients, and an outbreak management algorithm of daily testing for close contacts.

An outbreak management algorithm that tested the entire facility once with RATs on detection of a case had better outcomes than daily testing of only close contacts (13% reduction in deaths) or no contact testing (Table 1). Without any outbreak management testing there was minimal change in overall deaths, but a 6% increase in infections and a 23% decrease in diagnoses.

Routine surveillance testing was more effective than modifications to outbreak management testing algorithms. Twice weekly RAT testing of staff and residents reduced cumulative deaths by 61%, and reductions in cumulative deaths were still observed when only staff were tested, due to the greater number of staff in each RACF than residents (Table 1).

Minimal changes were seen when symptomatic testing was slightly increased or decreased; however extremely low symptomatic testing (i.e., 5% weekly probability) increased the number of deaths compared to the baseline by 94%.

### 3.4 Additional NPIs and vaccine rollout

**Figure 5:**
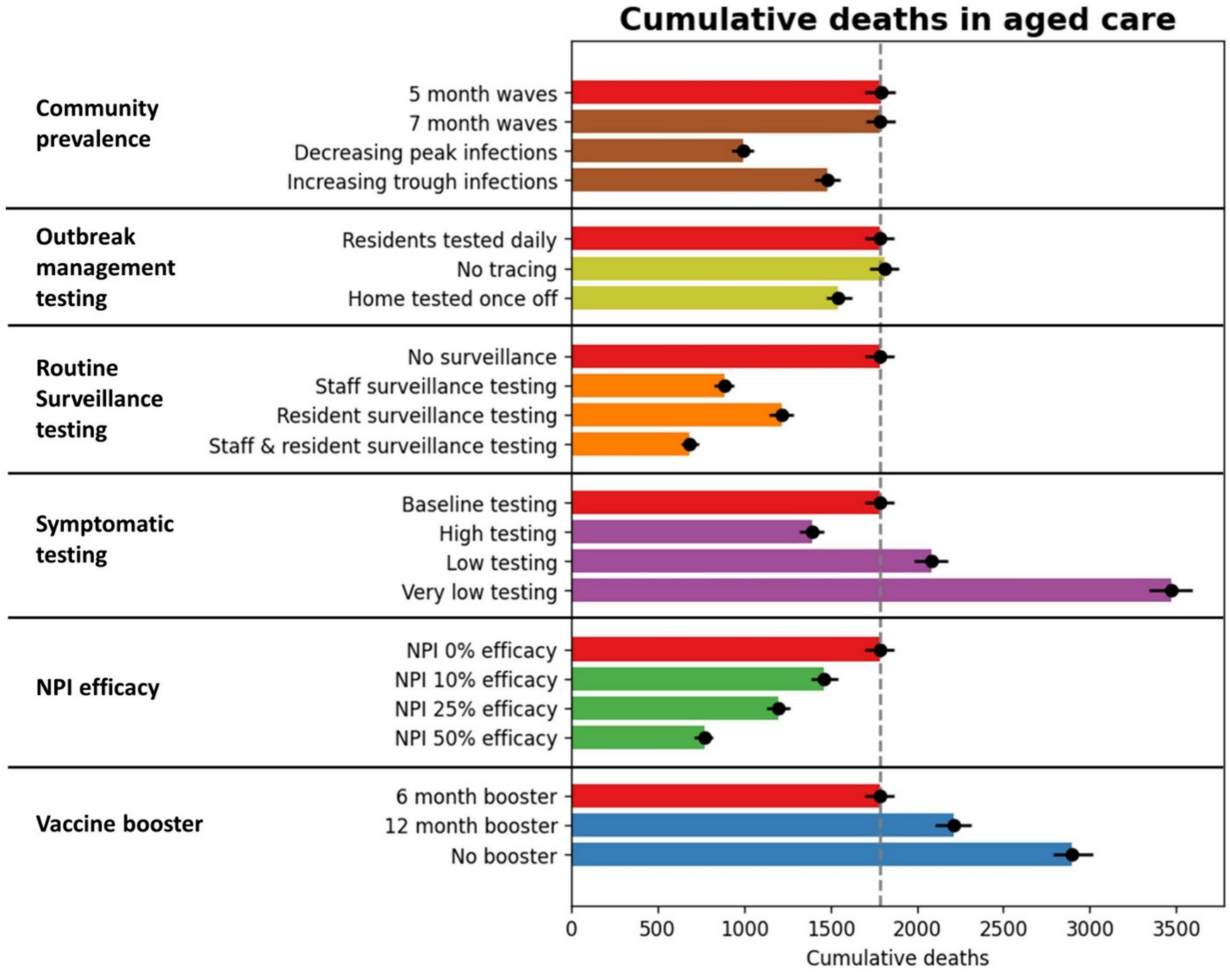
Cumulative outcomes between July 2023 and June 2024 for RACF scenarios. The red bars represent outcomes with baseline assumptions, and additional bars represent specific scenario outcomes.

Additional NPIs and increased vaccination had much greater impacts than the testing scenarios (Figure 5; Table 1). For example, NPIs that reduced transmission risk per contact in the model by 50% resulted in a 56% reduction in deaths compared to the baseline. The impact of additional NPIs within RACFs increased approximately linearly with NPI efficacy.

The baseline scenario assumed 6-monthly vaccine boosters for RACF residents, achieving 80% coverage. The non-linear waning of immunity (see Appendix) means that if boosters are delayed some protection is maintained for up to 12 months, but quickly declines beyond this. For example, when vaccine boosters were modeled at 12-monthly intervals, cumulative deaths over July 2023–June 2024 increased by 23%, and when no future vaccines were administered up to June 2024, cumulative deaths increased by 62%.

### 3.5 Sensitivity analyses

The model made assumptions on the number of incursions, incursion targets, and proportion of incursions to community prevalence. Sensitivity analyses were conducted to test each of these assumptions (Appendix C). The number of resident deaths increased (linearly) with number of initial incursions per simulation and number of incursions per 1,000 community infections, and were also higher when a greater proportion of incursions were among residents as opposed to staff (see Appendix A1).

## 4 Discussion

This analysis used an agent-based model to simulate COVID-19 outbreaks within RACFs in Victoria and assess the potential impact of a variety of interventions for reducing infections and associated mortality. This modeling identified community prevalence, vaccine rollout, NPIs, and surveillance testing as major factors influencing outcomes in RACFs, and how interventions could be best utilized to reduce mortality among residents.

Future community epidemic wave patterns are unknown but were identified as the biggest driver of outcomes within RACFs. A variety of patterns were tested, with the number of incursions into RACFs and outcomes within RACFs proportional to community infections over time. While community prevalence may be beyond the control of RACFs and their COVID-19 strategies, incursion-minimizing interventions such as routine visitor testing or visitor limits could have a large impact on RACF outcomes during periods of high community prevalence. In addition, the observed link between community prevalence and RACF deaths in the data highlights how interventions to reduce community-level prevalence can have flow-on benefits to RACFs.

Our results indicate that frequent vaccine booster campaigns will be integral in reducing adverse outcomes in RACFs. As the pandemic continues, the increased waning immunity and potential for decreased uptake from regular vaccination campaigns will leave residents considerably more vulnerable to severe outcomes following infection. The model suggests compared to 6-monthly booster campaigns, 12-monthly boosters resulted in moderately worse outcomes, but after more than 12-months without a booster the decrease in immunity was estimated to have major consequences within RACFs. It is therefore critical to ensure recency of vaccination is maintained for residents, particularly those who have missed a booster campaign for various reasons. As well as frequency, the timing of vaccine booster campaigns is another important policy consideration, because the relationship between community prevalence and RACF incursions means that residents are more likely to need the highest levels of immunity during community epidemic waves.

Before testing and mass vaccination were possible, NPIs such as density limits, masks, and ventilation were effective interventions for containing the spread of COVID-19 (35), with global studies investigating NPIs and their capability to reduce outbreak outcomes such as excess mortality rates in other settings (36–38). However, the direct impact on mortality from these studies are difficult to compare against this study due to the difference in baseline prevalence and methods. The results of this study showed that NPIs in RACFs, even with modest efficacy, can limit the size of outbreaks and lead to important reductions in resident cases and deaths. In our model we did not specific which NPIs were to be used and their mode of implementation, but rather tested different assumed effectiveness values (10, 25, 50%) that could likely be achieved through a combination of interventions. However, the type of NPIs that can be practically implemented over the longer term in RACFs requires some consideration; for example, NPIs restricting social interaction could severely impact quality of life (14, 39–41), while less restrictive NPIs such as increased ventilation, GUV, and mask wearing could be effective measures whilst still maintaining residents’ freedoms.

Testing of symptomatic residents, routine surveillance testing and outbreak management testing, followed by isolation of positive cases, were introduced early in the COVID-19 pandemic. In this analysis, varying levels of efficacy were displayed when different testing scenarios were implemented in RACFs. Routine surveillance testing effectively reduced resident deaths, by providing a means for pre-symptomatic or asymptomatic staff or residents to be identified and isolated from the rest of the facility. The baseline symptomatic testing was already estimated to be quite high within RACFs, but a scenario with very low symptomatic testing resulted in greatly increased deaths, confirming the importance of maintaining access to RATs and promotion of their use. Without any outbreak management testing there was an expected reduction in diagnoses, and little impact on infections and deaths. Conversely, enhancing outbreak management testing to include the entire facility and instead test everyone once rather than just close contacts daily had a greater impact reducing outbreak size and infections. These results collectively support the importance of testing and accompanying protocols with clear guidance on outbreak management testing within RACFs.

This work has some important limitations. Future epidemic waves are unknown, and results should not be interpreted as forecasts of outcomes, but as a way of comparing the relative effect of different interventions. Only existing variants are considered, while future waves could be heavily influenced by variants that are in circulation and vaccine effectiveness against these. Antiviral treatment coverage was considered as an input, however varying levels of treatment coverage could have important impact of reducing mortality. The model was calibrated to Victorian data, but reduced reporting of diagnoses over time and challenges in defining COVID-19 mortality among RACF residents means that some data may be incomplete, leading to uncertainty in model calibration (in unknown directions). The model does not simulate all individual aged care facilities in Victoria, but rather samples over facility characteristics, and therefore results represent average expected outcomes rather than outcomes for individual facilities. There is uncertainty in which interventions are already in place in the baseline scenario, due to facility-level heterogeneity regarding interventions and guidelines and policies that have changed over time. Additional scenarios may be considered such as varying levels of vaccine coverage or treatment coverage as well as therapeutic interventions (42). A future cost–benefit analysis could further strengthen understanding on what interventions may most effectively reduce mortality in RACFs.

## 5 Conclusion

A major driver of future outcomes in RACFs is likely to be community COVID-19 prevalence as a result of future COVID-19 epidemic waves. Maintaining high coverage of recent vaccination, minimizing incursions, regular testing and increasing NPIs can all have a major impact on cumulative deaths in aged care.

## Data availability statement

The original contributions presented in the study are included in the article/Supplementary material, further inquiries can be directed to the corresponding author.

## Author contributions

FM: Formal analysis, Methodology, Visualization, Writing – original draft. RS-D: Methodology, Writing – review & editing, Conceptualization. RA: Methodology, Software, Writing – review & editing, Conceptualization. DD: Methodology, Writing – review & editing. DW: Conceptualization, Data curation, Methodology, Writing – review & editing. IP: Data curation, Writing – review & editing. SM: Conceptualization, Writing – review & editing. MH: Writing – review & editing, Conceptualization. NS: Conceptualization, Formal analysis, Methodology, Supervision, Writing – original draft.

## References

[1] World Health Organisation. Who Coronavirus (COVID-19) Dashboard. Available at: https://covid19.who.int/

[2] Mathieu E Ritchie H Rodés-Guirao L Appel C Giattino C Hasell J. Coronavirus Pandemic (COVID-19). (2020). Available at: https://ourworldindata.org/coronavirus.

[3] Giattino C Ritchie H Ortiz-Ospina E Hasell J Rodés-Guirao L Roser M. Excess mortality during the Coronavirus pandemic (COVID-19). (2023). Available at: https://ourworldindata.org/excess-mortality-covid

[4] Barrett A. COVID-19 was the third leading cause of death in Australia last year. British Med. J. Pub. Group. (2023) 381:842. doi: 10.1136/bmj.p842, PMID: 37055068

[5] Levin AT Jylhava J Religa D Shallcross L. COVID-19 prevalence and mortality in longer-term care facilities. Eur J Epidemiol. (2022) 37:227–34. doi: 10.1007/s10654-022-00861-w, PMID: 35397704 PMC8994824

[6] Australian Institute of Health and Welfare. The impact of a new disease: COVID-19 from 2020, 2021 and into 2022. Canberra: AIHW (2022).

[7] Victorian Government. COVID-19 outbreaks in Australian residential aged care facilities - 28 April 2023. In: Department of Health and Aged Care, editor. (2023). Available at: https://www.health.gov.au/resources/publications/covid-19-outbreaks-in-australian-residential-aged-care-facilities-28-april-2023?language=en2023

[8] Storen R Corrigan N. COVID-19: a chronology of state and territory government announcements (up until 30 June 2020). In: Department of Parliamentary Services, editor. (2022). p. 72–86.

[9] Frazer K Mitchell L Stokes D Lacey E Crowley E Kelleher CC. A rapid systematic review of measures to protect older people in long-term care facilities from COVID-19. BMJ Open. (2021) 11:e047012. doi: 10.1136/bmjopen-2020-047012, PMID: 34663652 PMC8523961

[10] Department of Health and Aged Care. Priority access for Victoria residential aged care workers to get COVID-19 vaccination. In: Australian Government Department of Health, editor. Available at: https://www.health.gov.au/news/announcements/priority-access-for-victorian-residential-aged-care-workers-to-get-covid-19-vaccination2021

[11] Aged Care Quality and Safety Commission. COVID-19 oral antiviral treatments in Residential Aged Care Services. In: Aged Care Quality and Safety Commission, editor: Australian Government. (2022). Available at: https://www.agedcarequality.gov.au/sites/default/files/media/covid-19-oral-antiviral-treatments-in-residential-aged-care-services-fact-sheet.pdf

[12] Department of Health and Human Services. Plan for the Victorian Aged Care Sector. In: Department of Health and Human Services, editor.: Victoria State Government. (2020). p. 23–40.

[13] Chan DKY Mclaws M-L Forsyth DR. COVID-19 in aged care homes: a comparison of effects initial government policies had in the UK (primarily focussing on England) and Australia during the first wave (2021) 33. doi: 10.1093/intqhc/mzab033 PMC7989405 33677490

[14] Siette J Dodds L Seaman K Wuthrich V Johnco C Earl J. The impact of COVID‐19 on the quality of life of older adults receiving community‐based aged care. Australas. J. Ageing (2021) 40:84–9. doi: 10.1111/ajag.12924 33682315 PMC8250074

[15] Eden M Castonguay R Munkhbat B Balasubramanian H Gopalappa C. Agent-based evolving network modeling: a new simulation method for modeling low prevalence infectious diseases. Health Care Manag Sci. (2021) 24:623–39. doi: 10.1007/s10729-021-09558-0, PMID: 33991293 PMC8459606

[16] Perez L Dragicevic S. An agent-based approach for modeling dynamics of contagious disease spread. Int J Health Geogr. (2009) 8:50. doi: 10.1186/1476-072X-8-50, PMID: 19656403 PMC2729742

[17] Asgary A Blue H Solis AO McCarthy Z Najafabadi M Tofighi MA. Modeling COVID-19 outbreaks in long-term care facilities using an agent-based modeling and simulation approach. Int J Environ Res Public Health. (2022) 19:2635. doi: 10.3390/ijerph19052635, PMID: 35270344 PMC8910468

[18] Lasser J Zuber J Sorger J Dervic E Ledebur K Lindner SD. Agent-based simulations for protecting nursing homes with prevention and vaccination strategies. J R Soc Interface. (2021) 18:20210608. doi: 10.1098/rsif.2021.0608, PMID: 34932931 PMC8692030

[19] Lucia-Sanz A Magalie A Rodriguez-Gonzalez R Leung CY Weitz JS. Modeling shield immunity to reduce COVID-19 transmission in long-term care facilities. Ann Epidemiol. (2023) 77:44–52. doi: 10.1016/j.annepidem.2022.10.013, PMID: 36356685 PMC9639409

[20] Gaddis MD Manoranjan VS. Modeling the spread of COVID-19 in enclosed spaces. J. Math. Comput. Appl. (2021) 26:79.

[21] Australian Institute of Health and Welfare. (2023). Providers, services and places in aged care 2023. Available at: https://www.gen-agedcaredata.gov.au/Topics/Providers,-services-and-places-in-aged-care.

[22] Department of Health. Personal Communication with Department of Health. In: Burnet Institute, editor. (2022).

[23] Kerr CC Stuart RM Mistry D Abeysuriya RG Rosenfeld K Hart GR. Covasim: an agent-based model of COVID-19 dynamics and interventions. PLoS Comput Biol. (2021) 17:e1009149. doi: 10.1371/journal.pcbi.1009149, PMID: 34310589 PMC8341708

[24] Institute for Disease Modelling. Covasim model GitHub Repository 2021. (2021). Available from: https://github.com/InstituteforDiseaseModeling/covasim.

[25] Scott N Abeysuriya RG Delport D Sacks-Davis R Nolan J West D. COVID-19 epidemic modelling for policy decision support in Victoria, Australia 2020-2021. BMC Public Health. (2023) 23:988. doi: 10.1186/s12889-023-15936-w, PMID: 37237343 PMC10219801

[26] Scott N Palmer A Delport D Abeysuriya R Stuart RM Kerr CC. Modelling the impact of relaxing COVID-19 control measures during a period of low viral transmission. Med J Aust. (2021) 214:79–83. doi: 10.5694/mja2.50845, PMID: 33207390 PMC7753668

[27] Abeysuriya RG Delport D Stuart RM Sacks-Davis R Kerr CC Mistry D. Preventing a cluster from becoming a new wave in settings with zero community COVID-19 cases. BMC Infect Dis. (2022) 22:232. doi: 10.1186/s12879-022-07180-1, PMID: 35255823 PMC8899797

[28] Abeysuriya RG Sacks-Davis R Heath K Delport D Russell FM Danchin M. Keeping kids in school: modelling school-based testing and quarantine strategies during the COVID-19 pandemic in Australia. Front Public Health. (2023) 11:1150810. doi: 10.3389/fpubh.2023.1150810, PMID: 37333560 PMC10272722

[29] Department of Health. Management of Acute Respiratory Infection outbreaks, including COVID-19 and influenza, in residential care facilities (RCFs). In: Department of Health, editor. Available at: https://www.health.vic.gov.au/publications/management-of-acute-respiratory-infection-outbreaks-including-covid-19-and-influenza2022. p. 26, 35, 9–40, 2–4.

[30] Australian Government. National COVID-19 Health Management Plan for 2023. In: Department of Health and Aged Care, editor. (2023). Available at: https://www.health.gov.au/resources/publications/national-covid-19-health-management-plan-for-20232022. p. 13–4.

[31] Department of Health and Aged Care. COVID-19 vaccine rollout updates - 6 October 2022. In: Department of Health and Aged Care, editor. (2022). Available at: https://www.health.gov.au/resources/publications/covid-19-vaccine-rollout-update-6-october-2022?language=en2022.

[32] Esmaeili ED Azizi H Sarbazi E Khodamoradi F. The global case fatality rate due to COVID-19 in hospitalized elderly patients by sex, year, gross domestic product, and continent: a systematic review, meta-analysis, and meta-regression. New Microbes New Infect. (2023) 51:101079. doi: 10.1016/j.nmni.2022.101079, PMID: 36618974 PMC9811917

[33] Mendez-Brito A El Bcheraoui C Pozo-Martin F. Systematic review of empirical studies comparing the effectiveness of non-pharmaceutical interventions against COVID-19. J Infect. (2021) 83:281–93. doi: 10.1016/j.jinf.2021.06.018, PMID: 34161818 PMC8214911

[34] Kumar A Sagdeo A Sagdeo PR. Possibility of using ultraviolet radiation for disinfecting the novel COVID-19. Photodiagn Photodyn Ther. (2021) 34:102234. doi: 10.1016/j.pdpdt.2021.102234, PMID: 33639320 PMC7903903

[35] Perra N. Non-pharmaceutical interventions during the COVID-19 pandemic: a review. Phys. Rep. (2021) 913:1–52. doi: 10.1016/j.physrep.2021.02.001 33612922 PMC7881715

[36] Ghosal S Bhattacharyya R Majumder M. Impact of complete lockdown on total infection and death rates: a hierarchical cluster analysis. Diabetes Metab Syndr. (2020) 14:707–11. doi: 10.1016/j.dsx.2020.05.026, PMID: 32426062 PMC7227592

[37] Cho S-W. Quantifying the impact of nonpharmaceutical interventions during the COVID-19 outbreak: the case of Sweden. J. Econom. (2020) 23:323–44. doi: 10.1093/ectj/utaa025

[38] Stokes J Turner AJ Anselmi L Morciano M Hone T. The relative effects of non-pharmaceutical interventions on wave one Covid-19 mortality: natural experiment in 130 countries. BMC Public Health. (2022) 22:1113. doi: 10.1186/s12889-022-13546-6, PMID: 35659646 PMC9165709

[39] Armitage R Nellums LB. COVID-19 and the consequences of isolating the elderly. Lancet Public Health. (2020) 5:e256. doi: 10.1016/S2468-2667(20)30061-X, PMID: 32199471 PMC7104160

[40] Thomas S Bolsewicz K Latta R Hewitt J Byles J Durrheim D. The impact of public health restrictions in residential aged care on residents, families, and staff during COVID-19: getting the balance right. J Aging Soc Policy. (2022) 10:1–20. doi: 10.1080/08959420.2022.2110802, PMID: 35946918

[41] Brydon A Bhar S Doyle C Batchelor F Lovelock H Almond H. National Survey on the impact of COVID-19 on the mental health of Australian residential aged care residents and staff. Clin Gerontol. (2022) 45:58–70. doi: 10.1080/07317115.2021.1985671, PMID: 34634217

[42] Callender LA Curran M Bates SM Mairesse M Weigandt J Betts CJ. The impact of pre-existing comorbidities and therapeutic interventions on COVID-19. Front Immunol. (2020) 11:1991. doi: 10.3389/fimmu.2020.01991, PMID: 32903476 PMC7437504

