# Supplementary material for: COVID-19 outbreaks in residential aged care facilities: an agent-based modeling study
Source: Front Public Health. 2024 May 21;12:1344916. doi: 10.3389/fpubh.2024.1344916 (PMC11148262; doi:10.3389/fpubh.2024.1344916)
Supplement: Supplementary file 1 [file Data_Sheet_1.docx]

**Supplementary material**

1. Model inputs
   1. Model population

RACF characteristics used for the model are in Table 1. The distributions of residents and staff per RACF were fitted to 2022 Victorian line listed aged care data. The number of resident-resident, staff-resident, and staff-staff interactions as well as infectiousness between contacts were determined through calibration as discussed in sections B.2 and B.3.

Table 1: RACF assumptions. Values taken from data or determined through calibration.

| **Parameter** | **Distribution** | **Inputs** | **Note** |
| --- | --- | --- | --- |
| Number of residents per RACF | Poisson | Mean: 62  Minimum: 40 | From Victoria RACF data |
| Number of staff per RACF | Normal | Mean: 91  Standard deviation: 49 | From Victoria RACF data |
| Resident-resident contacts | Poisson | Mean: 5 | Determined through calibration |
| Staff-resident contacts | Poisson | Mean: 5 | Determined through calibration |
| Staff-staff contacts | Poisson | Mean: 5 | Determined through calibration |
| Transmission risk relative to resident-resident | Constant value | Reference |  |
| Resident-staff: Transmission risk relative to resident-resident | Constant value | Value: 0.87 | Determined through calibration |
| Staff-staff: Transmission risk relative to resident-resident | Constant value | Value: 0.66 | Determined through calibration |

Severity of outcomes following infection needed to be adjusted for RACF residents compared to the general population due to the high prevalence of comorbidities. This was done by scaling the age-specific estimates [1] by a factor (1.5), determined though calibration to case fatality rates. Table 2 shows calibrated values for outcomes for people 80-89 and 90+ years who have no prior immunity through either vaccination or exposure. Outcomes are then modified for people receiving vaccinations.

Table 2: Prognosis probabilities for people with no prior immunity (vaccination or infection): determined through calibration.

| **Age bracket** | **Pr(Symptomatic \| infection)** | **Pr(Severe \| infection)** | **Pr(Critical \| infection)** | **Pr(Death \| infection)** |
| --- | --- | --- | --- | --- |
| 80-89 | 1 | 0.825 | 0.4455 | 0.182415 |
| 90+ | 1 | 0.825 | 0.4455 | 0.182415 |

Each agent is assigned an age and a resident or staff status, with staff being aged 18-65 and residents aged 80+. The model does not disaggregate by sex or ethnicity due to insufficient data.

- 1. Testing & Contact tracing

Residents and staff in the model have a daily probability of testing if they are symptomatic and a daily probability of testing if they are asymptomatic (i.e. they might be symptomatic for another disease that prompts testing to detect asymptomatic COVID-19). If someone experiences a severe infection, they are assumed to be tested automatically.

Following detection of a case, a contact tracing algorithm is initiated. The baseline assumption is that once a case is identified all residents in the home test every day for 7 days, but an alternate of only a single test is included as well.

Routine surveillance testing is also included with the option of twice weekly testing for either staff, residents, or both.

The model assumes PCR (87% sensitivity [2]) testing until January 2022 before moving to RAT testing (77.3% sensitivity [2, 3]). All tests have an assumed 0-day turnover from taking the test to receiving the result.

- 1. Quarantine & Isolation

Once a staff or resident tests positive, they are assumed to isolate for 7 days. No quarantine of close contacts is included as per RACF protocols from 2022.

- 1. Vaccination

In the model, vaccination acts to reduce the probability of acquiring an infection when a contact occurs with an infectious case, as well as the probability of developing symptoms (both mild and severe) for people who are vaccinated and become infected.

Individuals are modelled to have a level of “neutralizing antibodies” (NAbs). NAbs can be acquired through either vaccination or infection, with different doses of different vaccines leading to different levels of NAbs. NAbs are then assumed to wane over time following an exponential function. A separate logistic relationship is modelled that relate a person’s NAb levels to estimates for protection against infection, symptoms and severe disease [4]. For the Pfizer vaccine, the induced peak NAb levels following vaccination and rate of waning were calibrated to align with UK SAGE estimates [5] for protection against infection, symptoms and severe disease from the Delta variant (Figure 1**,** Table 3), and adjusted down by 95% based on estimates of the immune escape of Omicron variants. The peak NAbs following a third dose were calibrated to produce the increase in protection from the published literature [6, 7] (Table 3), and further booster doses were assumed equivalent to the third dose.


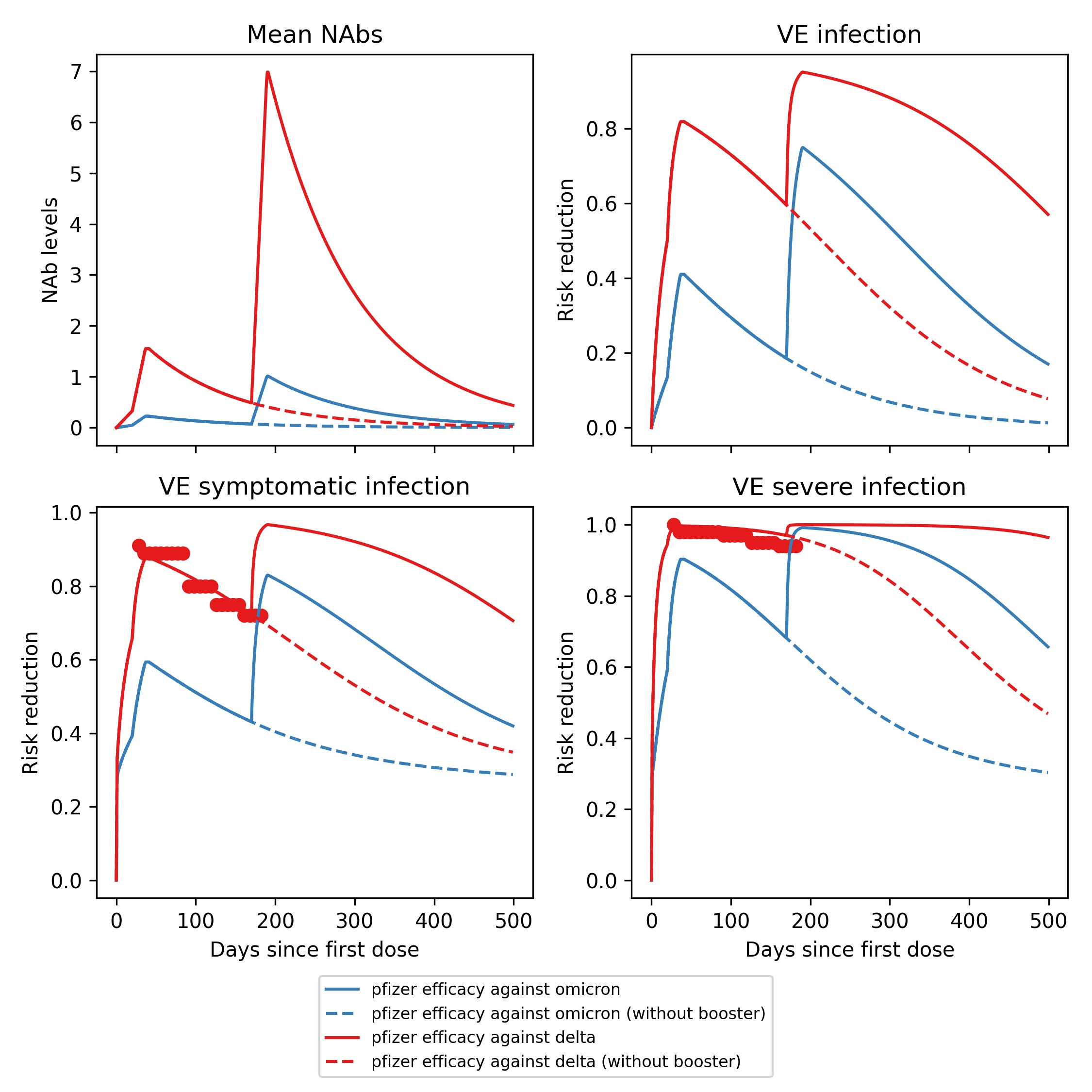


Figure 1: Estimated efficacy of Pfizer vaccines against Delta and Omicron variants over time. Time between consecutive Pfizer doses are assumed to be 3 weeks (second dose) and 5 months (third dose). Dots correspond to estimated efficacy values from UK SAGE, Sep 2021 [5].

Table 3: Peak vaccine efficacy against Delta and Omicron, based on implementation in Figure 1, and UK SAGE, Sep 2021 estimates [5].

| **Vaccine** | **Variant** | **Dose** | **Peak protection against infection** | **Peak protection against symptomatic infection** | **Peak protection against severe disease** |
| --- | --- | --- | --- | --- | --- |
| Pfizer | Delta | 1 | 35% | 55% | 86% |
|  |  | 2 | 82% | 88% | 100% |
|  |  | 3 | 94% | 94% | 99.9% |
|  | Omicron | 1 | 13% | 40% | 60% |
|  |  | 2 | 41% | 60% | 90% |
|  |  | 3 | 75% | 83% | 99% |


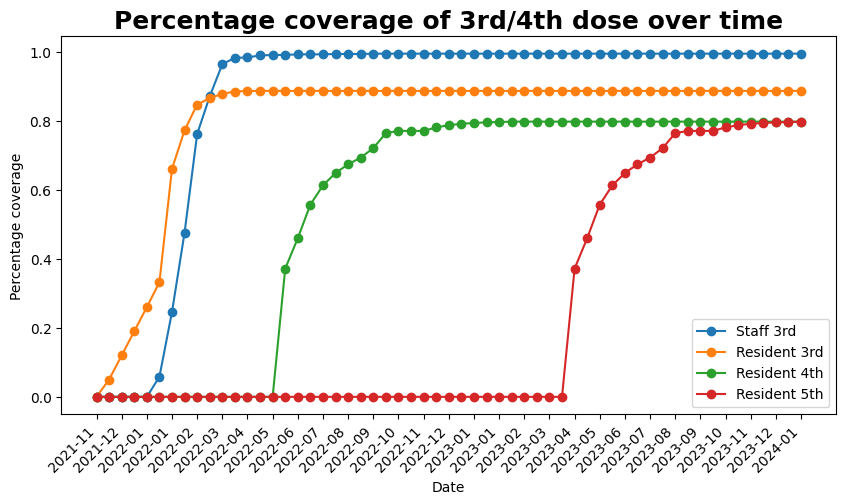


Figure 2: Vaccine booster coverage over time for Victorian RACF staff and residents. Resident third dose data for November/December 2021 was not available but estimated as a linear scale-up.

- 1. Treatment

Treatment is modelled by reducing the probability of death given infection and severity given infection. Treatment is assumed to be with Molnupiravir (Lagevrio) with an efficacy of 50% against deaths [8] and 45% against becoming severe [9].

Treatment effectiveness at a population-level is very dependent on how it is targeted to people who are at highest risk. A continuous function is used to relate treatment coverage to reduction in severe outcomes (Figure 4). For example, a linear function would assume everyone in each age group is at equal risk of severe outcomes, meaning a 30% treatment coverage would lead to a 30% reduction in severe outcomes. However this assumption would underestimate the impact of low (but targeted) treatment numbers, and overestimate the incremental impact of increasing treatment coverage. Therefore, the resident population is assigned a percentage representing how many people are likely to be high risk (Figure 4) [8]. By broadly classifying everyone as either high risk or low risk, this can encapsulate the potential greater effects of low treatment numbers, and gives a continuous function which captures the decline in treatment impact with scale (Figure 4). In the model, treatment is only applied to residents in RACFs.

Time varying treatment coverage is applied starting in February 2022 (Figure 3). A Pharmaceutical Benefits Scheme (PBS) treatment rollout was tracked in aged care over time with data on the treatment coverage starting in March 2022. An additional 20k doses were supplied by the National Medical Stockpile (NMS) where timing is unknown, this is approximated by a 20% treatment coverage between February and March.


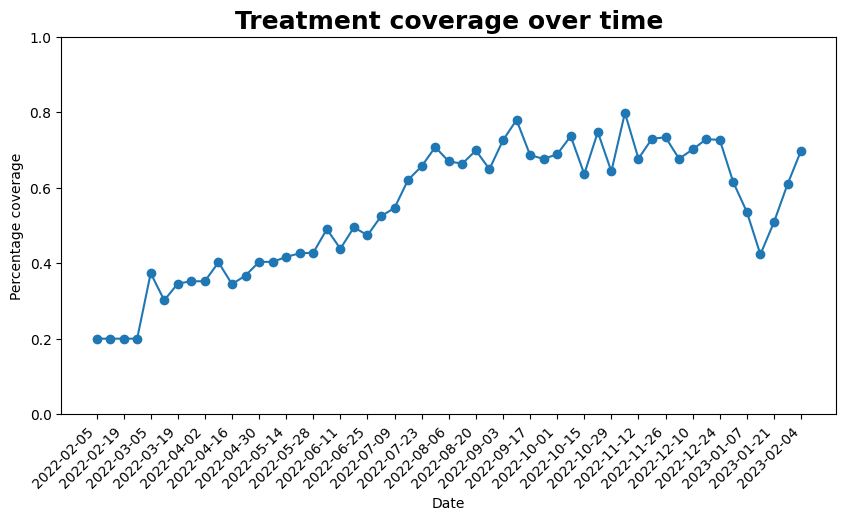


Figure 3: Resident treatment coverage over time taken from Victoria RACF data. Coverage between February and March assumed 20% from early NMS allocation.


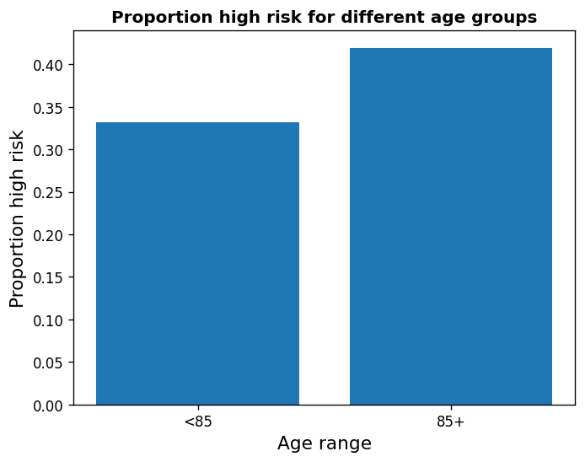

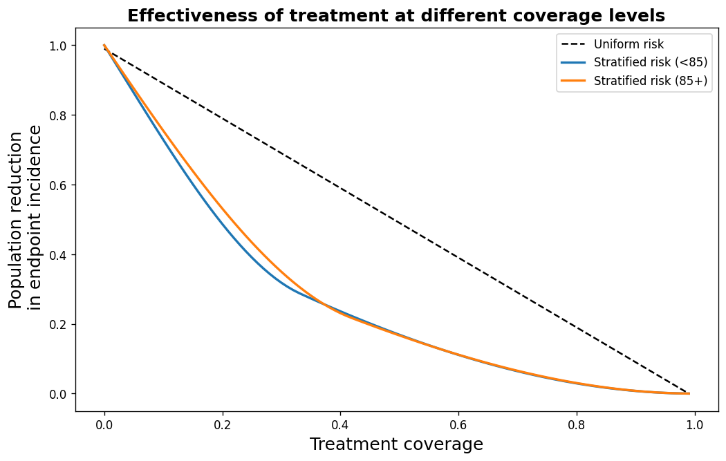


Figure 4: (Left) Proportion of those at high risk for < 85 and 85+ age categories [8]. (Right) Effectiveness of treatment at different coverage levels for those less than 85 and great than 85 years old, a linear (uniform) relationship is presented to display the differences in outcomes if non stratified risk was assumed.

- 1. Variants

The variant that is modelled for each incursion is determined from the distribution of variants in circulation in Victoria at the time. Each variant has assumed infectiousness and immune escape characteristics derived from independent modelling studies that have fitted these variants to Victorian sequencing and wastewater data over time (see Appendix B.4). The relative infectiousness and immune escape of each variant in Table 4 is compared to Omicron BA.1, and the vaccine efficacy^1^ is assumed to be the same against all Omicron variants.

Table 4: Infectiousness and immune escape values for different variants.

| **Variant** | **Infectiousness** | **Immune escape from NAbs^1^ through past infection (relative to BA.1)** | **Immune escape from NAbs****^[[1]](#footnote-2)^ through vaccination** |
| --- | --- | --- | --- |
| Omicron BA.1 | 1 | 1 | 0.05 |
| BA.2 | 1.35 | 1 | 0.05 |
| BA.4/BA.5 | 1.5525 | 0.45 | 0.05 |
| XBF | 1.5525 | 0.2 | 0.05 |


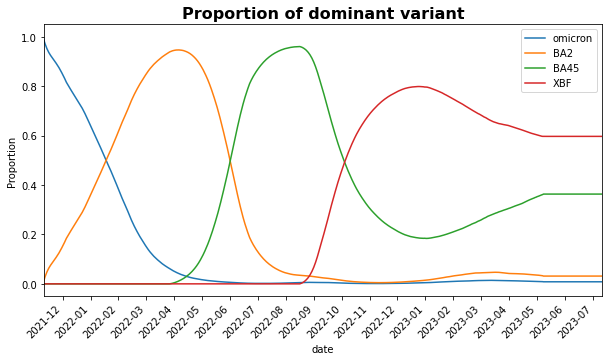


Figure 5: Timeseries graph representing proportion of dominant variant between December 2021 and July 2023. Variant proportion estimates taken from independent whole of population model (Appendix B.4).

- 1. Other model parameters

The baseline assumption for future community prevalence assumed that similar epidemic wave patterns would continue into the future, as had occurred over 2022. As with the proportional distribution of different variants, and variant characteristics, the 2022 infection prevalence was derived from independent modelling studies (see Appendix B.4). The result was a baseline of a sinusoidal epidemic wave function with 5-monthly waves and peaks, like that of the XBB.1.5 peak infections.


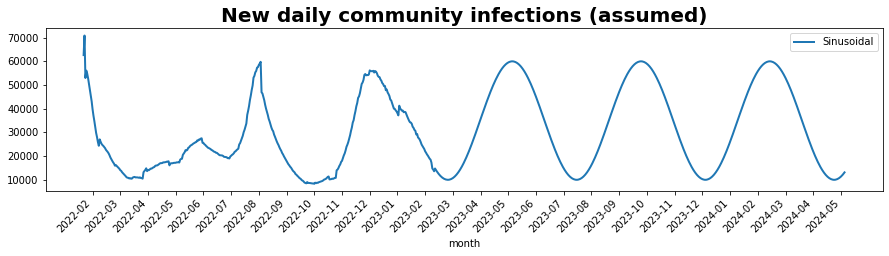


Figure 6: Community prevalence assumptions. The baseline future wave assumption is chosen to be sinusoidal with a 5-month period and a magnitude like the XBB.1.5 peak.

| **Date** | **Policy** |
| --- | --- |
| 2022-02-01 | Shift from PCR to RAT testing |
| 2022-03-01 | Increase in testing by 30% after increased quantity of RATs |
| 2022-06-01 | Reverse 30% testing increase (decrease in testing back to baseline) |
| 2022-04-23 | Quarantine requirements changed to 5-days |

1. Calibration
   1. Calibration step 1: Outbreak size and duration

The model was calibrated to outbreak duration and outbreak size among resident and staff in Victorian RACFs for outbreaks occurring in December 2022. The calibration month was arbitrary but had to be selected because different months have different immunity profiles and intervention coverage, which influence outbreaks. Calibration was achieved by adjusting the mean contacts per day and relative risk of transmission between contacts within RACFs (for staff-staff, staff-resident and resident-resident contacts), as well as risk mitigation upon detection of first case. Values were chosen to match the distribution of resident and staff outbreak size (Figure 7) and outbreak duration (Figure 8). The calibration is based on diagnoses (rather than infections) as this is what is recorded; in the model case ascertainment is imperfect and is an outcome of testing inputs.


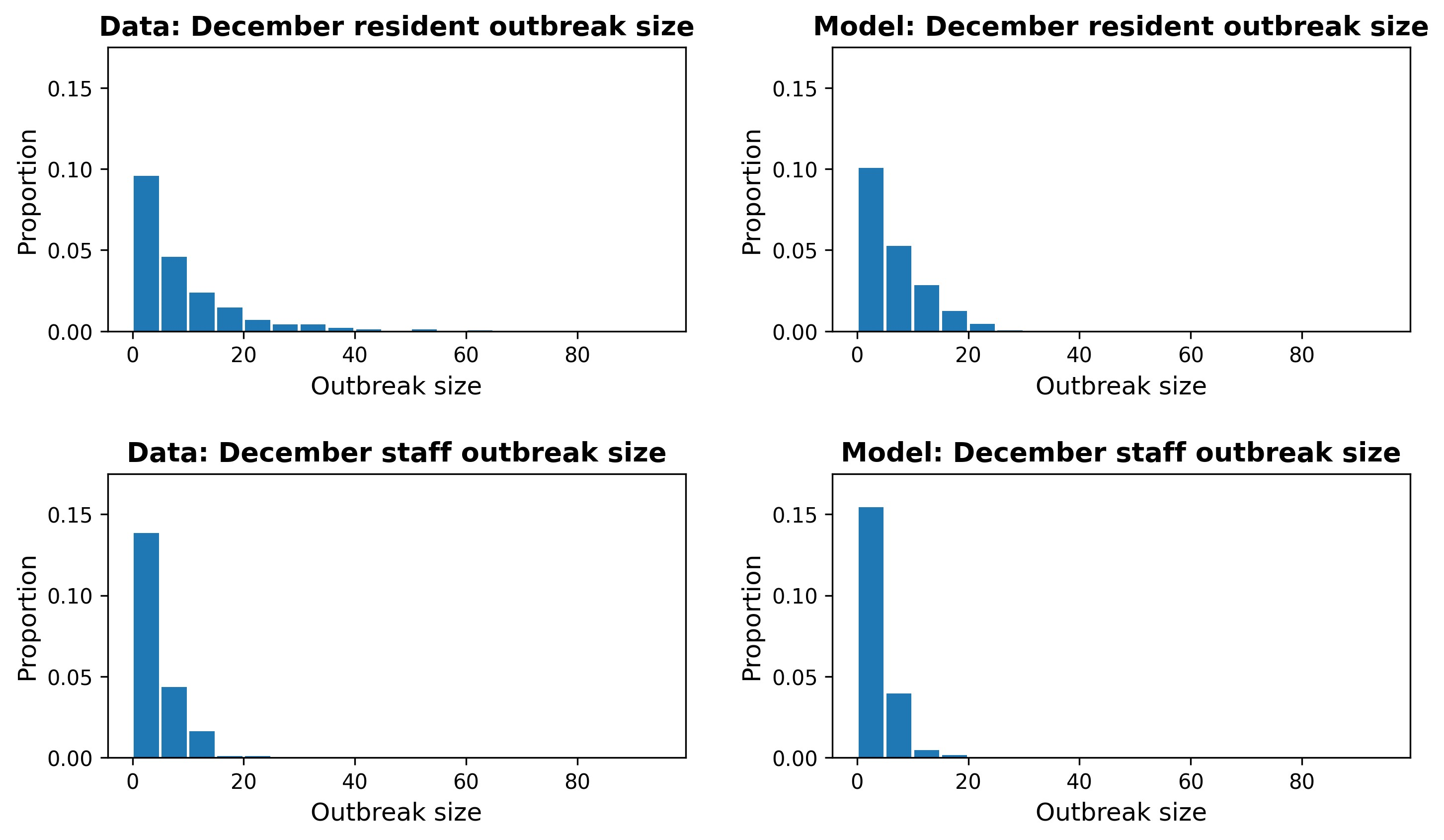


Figure 7: Model calibration to outbreak size. (Left) Resident and staff outbreaks size from Victoria RACF data in December 2022. (Right) distribution of resident and staff infections over 2000 simulation, only outbreaks with 1 or more diagnoses were considered.


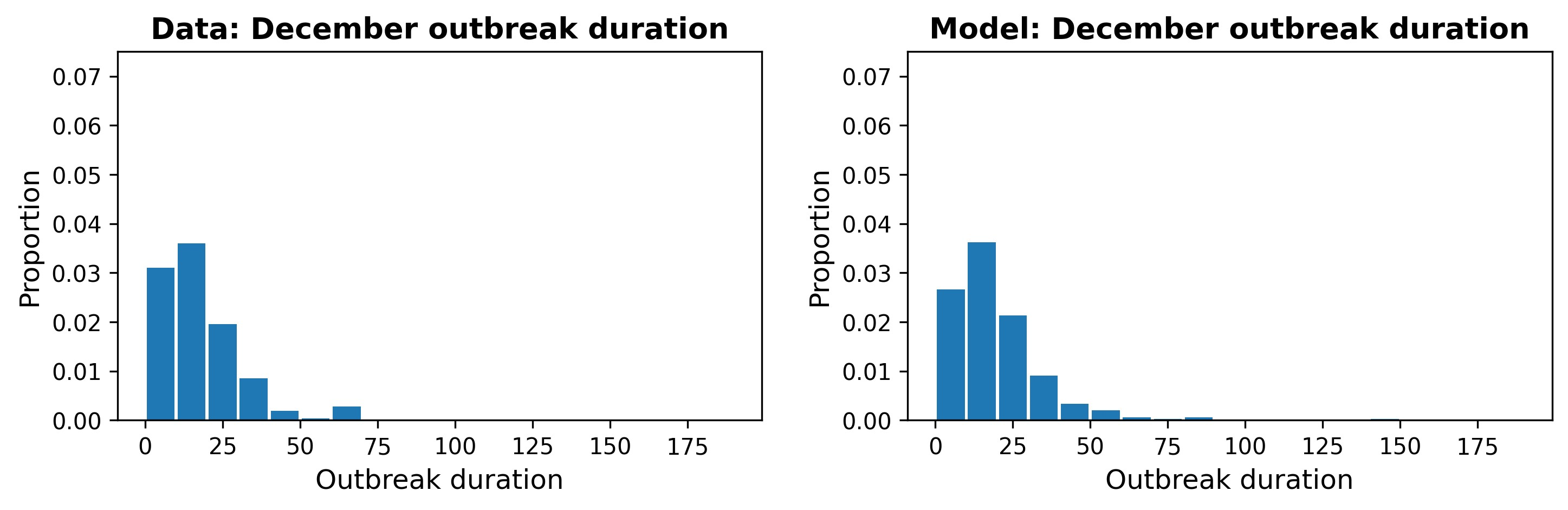


Figure 8: Model calibration outbreak duration. (Left) Outbreak duration from Victorian RACF data in December 2022. (Right) distribution of outbreak durations over 2000 simulations, only outbreaks with 1 or more diagnoses were considered.

- 1. Calibration step 2: Case fatality rate

The model was calibrated to case fatality rate (COVID-19 deaths / diagnoses) within RACFs in December 2022 based on Victoria RACF data. In December 2022 approximately 5% of aged care residents diagnosed with COVID-19 died from the disease. The probability of death given *infection* for RACF residents was adjusted such that the model reproduced case fatality data (deaths / diagnoses) in December for simulated outbreaks.

The modelled case fatality rate varies over time due to treatment and vaccination coverage and waning immunity; Figure 9 shows how the model compares to the data for each month, which includes the impacts of treatment, vaccination, and waning immunity. February 2022 is likely to be an outlier as there was an Australia-wide shortage of diagnostic tests, which may lead to lower diagnoses rates and higher case fatality rate in that month.


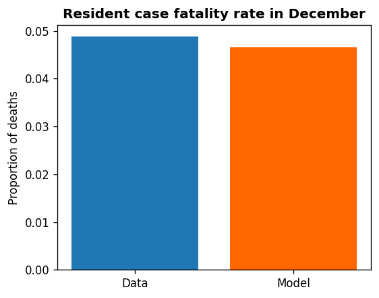


Figure 9: Probability of death given infection compared to Victorian RACF data in December 2022.


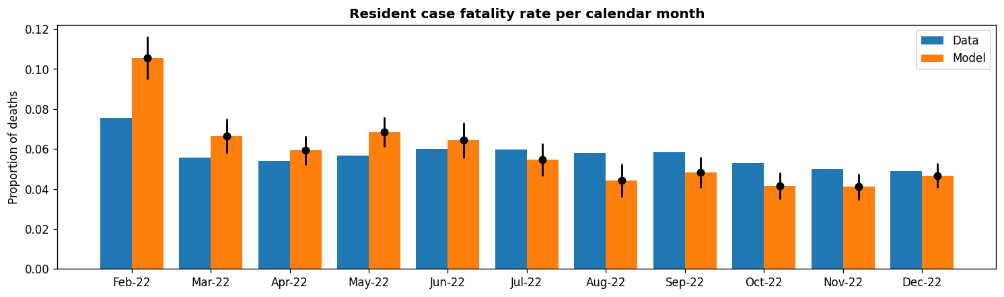


Figure 10: Probability of death given infection compared to Victorian RACF data between February 2022 - December 2022.

- 1. Calibration step 3: Incursion rate

Once the model was calibrated to outcomes *per incursion*, it needed to be calibrated to incursion rates into RACFs from the community (i.e. incursions per 1000 community infections). This involved two steps: first estimating the number of community infections over time (see Appendix B.4), and second estimating the relationship between community infections and number of incursions.

Data for diagnoses and deaths in RACFs were assigned to calendar months based on the date of first diagnosis of the outbreak they were linked to.

For each calendar month, incursion-outcome libraries were created by simulating 1000 incursions based on the conditions in the model during that month (i.e. vaccine and treatment coverage, exposure-acquired immunity, policies) (Figure 11). For a given assumption about the number of monthly incursions, the incursion-outcome libraries could then be sampled this many times to produce an estimate for total number of deaths and diagnoses. Repeated sampling was used to generate uncertainty intervals for outcomes for each month.

The model was then calibrated to total number of deaths and diagnoses for each month between February 2022 and October 2022 by estimating a rate of incursion into RACFs for each month that achieved the best fit (data vs sampled incursion-outcome libraries). The number of incursions into aged care were assumed to be proportional to community infections, but different relationships were tested and resulted in poorer fits (constant number of incursions each month, gradually increasing incursions per month, and incursions proportional to community diagnoses).

The result was an estimate of 3 incursions per 1000 community infections, which achieved the fit show in . There is known to be underreporting of outcomes in Feb 2022 in the data, due to shortages of testing at the time, which is why the model produces very different outcomes. For the later months (Aug-Oct 2022), data reporting was less complete, partially explaining the discrepancies between the model outcomes. Sensitivity analyses were used to test the impact of 1 incursion per 1000 community infections and 2 incursions per 1000 community infections (see Appendix C.3).


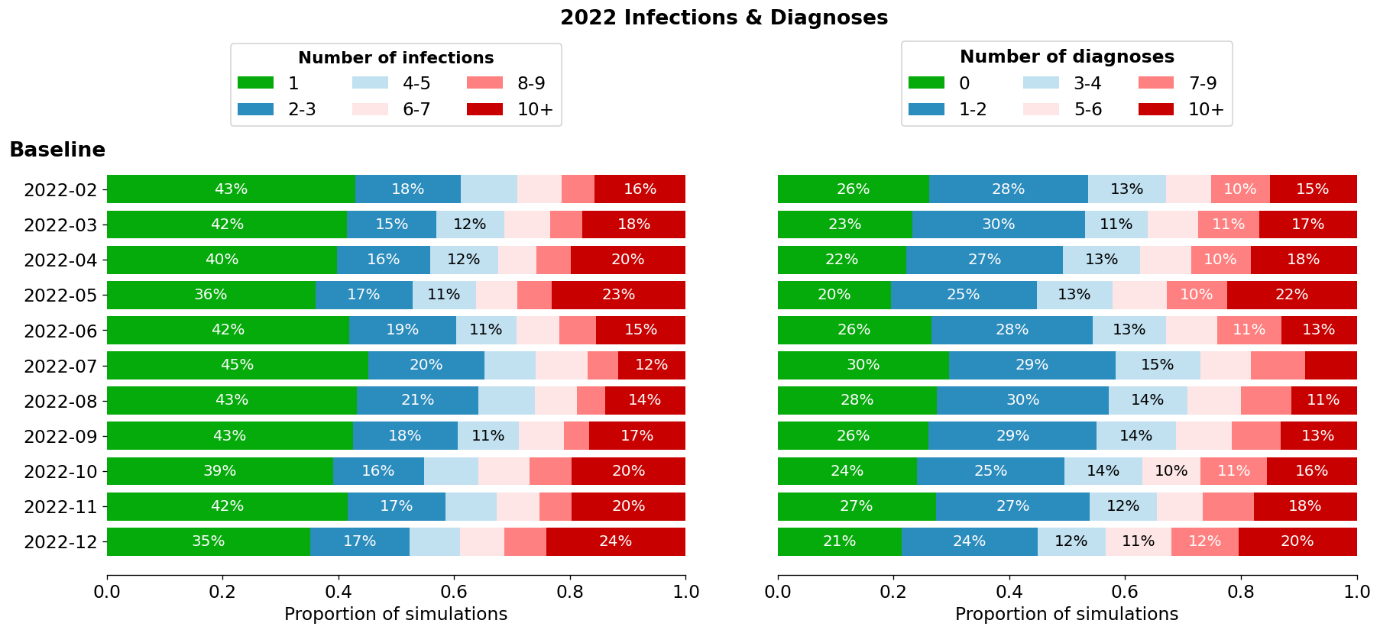


Figure 11: Incursion outcome library infection and diagnoses distribution. Horizontal bar plot showing aged care outbreak distribution for each month from incursion outcome library.


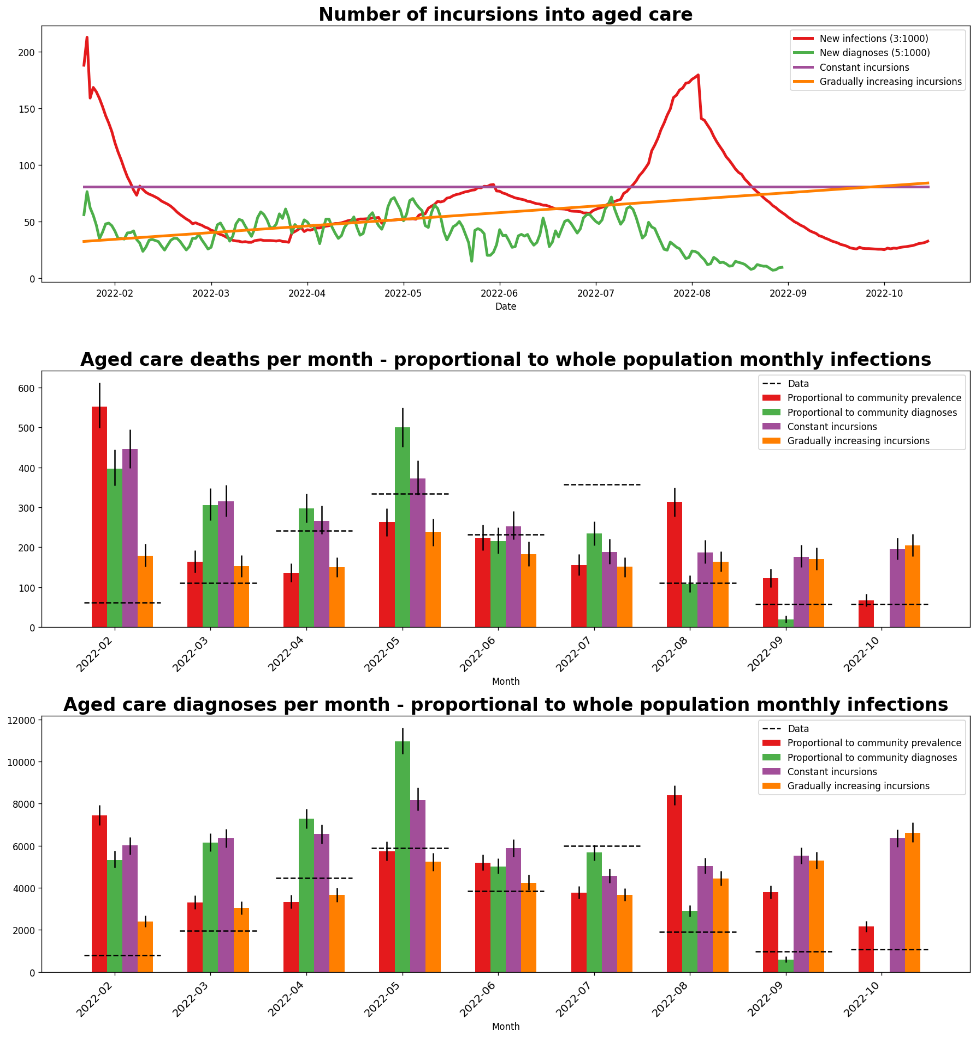


Figure 12: Total deaths/diagnoses in aged care in 2022 per month for different incursion relationships. The top graph represents the total number incursions into RACFs over time for different relationships. Total number of aged care diagnoses and deaths per month from Victorian RACF data are represented by the black dotted line. Due to potential underreporting and mandatory test reporting ceased in late 2022, community diagnoses were not a good indicator of infections in the community [Cite].

Table 5: Distribution of infections with uncertainty (aggregate among staff + residents) for incursions occurring in each month between February 2022 and December 2022.

| Number of infections | 1 | 2-3 | 4-5 | 6-7 | 8-9 | 10+ |
| --- | --- | --- | --- | --- | --- | --- |
| 2022-02 | 43.0%  (39.6% - 46.6%) | 18.2%  (15.4% - 21.4%) | 9.6%  (7.4% - 11.7%) | 7.7%  (5.8% - 9.8%) | 5.7%  (4.1% - 7.6%) | 15.7%  (13.0% - 18.4%) |
| 2022-03 | 41.5%  (37.8% - 45.1%) | 15.4%  (12.5% - 18.6%) | 11.7%  (9.3% - 14.3%) | 8.0%  (6.0% - 10.0%) | 5.5%  (3.8% - 7.4%) | 17.9%  (15.2% - 20.8%) |
| 2022-04 | 40.0%  (36.5% - 43.8%) | 16.0%  (13.4% - 18.5%) | 11.7%  (9.4% - 13.7%) | 6.6%  (4.8% - 8.4%) | 5.8%  (4.3% - 7.6%) | 20.0%  (16.9% - 22.9%) |
| 2022-05 | 36.1%  (32.7% - 39.5%) | 16.9%  (14.2% - 19.9%) | 11.2%  (8.6% - 13.6%) | 7.1%  (5.4% - 8.8%) | 6.0%  (4.3% - 7.8%) | 23.2%  (20.1% - 26.0%) |
| 2022-06 | 41.7%  (38.0% - 45.1%) | 18.5%  (15.8% - 21.2%) | 10.5%  (8.3% - 12.6%) | 7.3%  (5.4% - 9.3%) | 6.5%  (4.8% - 8.1%) | 15.4%  (13.0% - 17.8%) |
| 2022-07 | 45.3%  (41.3% - 48.8%) | 20.1%  (17.0% - 23.4%) | 8.8%  (6.9% - 10.9%) | 9.0%  (6.9% - 11.3%) | 5.3%  (3.8% - 7.3%) | 11.6%  (9.2% - 13.9%) |
| 2022-08 | 43.0%  (39.1% - 46.7%) | 21.0%  (18.1% - 23.7%) | 9.8%  (7.9% - 11.9%) | 7.2%  (5.4% - 9.5%) | 4.9%  (3.5% - 6.5%) | 13.9%  (11.3% - 16.7%) |
| 2022-09 | 42.5%  (39.0% - 46.1%) | 18.1%  (15.7% - 20.7%) | 10.6%  (8.3% - 13.2%) | 7.9%  (5.9% - 10.1%) | 4.3%  (2.8% - 5.9%) | 16.7%  (13.9% - 19.6%) |
| 2022-10 | 39.0%  (35.7% - 42.3%) | 15.8%  (13.2% - 18.2%) | 9.3%  (7.3% - 11.3%) | 8.9%  (6.9% - 10.9%) | 7.1%  (5.5% - 8.9%) | 19.7%  (16.9% - 22.3%) |
| 2022-11 | 41.7%  (37.8% - 45.1%) | 16.9%  (14.3% - 19.7%) | 9.0%  (7.1% - 11.0%) | 7.4%  (5.8% - 9.2%) | 5.4%  (3.7% - 7.2%) | 19.7%  (17.0% - 22.5%) |
| 2022-12 | 35.1%  (31.8% - 38.5%) | 17.1%  (14.3% - 20.0%) | 8.8%  (6.8% - 10.8%) | 7.7%  (5.5% - 9.6%) | 7.3%  (5.5% - 8.9%) | 24.0%  (21.0% - 26.8%) |

Table 6: Distribution of infections with uncertainty (aggregate among staff + residents) for incursions occurring in each month between February 2022 and December 2022.

| Number of diagnoses | 0 | 1-2 | 3-4 | 5-6 | 7-9 | 10+ |
| --- | --- | --- | --- | --- | --- | --- |
| 2022-02 | 26.2%  (23.3% - 29.3%) | 27.4%  (24.7% - 30.2%) | 13.4%  (11.1% - 16.1%) | 7.6%  (5.9% - 9.6%) | 10.2%  (7.9% - 12.3%) | 15.1%  (12.7% - 17.6%) |
| 2022-03 | 23.3%  (20.2% - 26.4%) | 29.9%  (26.4% - 33.5%) | 10.7%  (8.5% - 13.2%) | 8.7%  (6.5% - 10.9%) | 10.6%  (8.1% - 12.9%) | 16.8%  (13.7% - 19.8%) |
| 2022-04 | 22.2%  (19.0% - 25.1%) | 27.0%  (23.8% - 30.3%) | 13.5%  (11.0% - 16.4%) | 8.7%  (6.7% - 11.0%) | 10.4%  (7.8% - 12.6%) | 18.4%  (15.3% - 21.4%) |
| 2022-05 | 19.5%  (16.4% - 22.8%) | 25.3%  (22.3% - 28.5%) | 13.1%  (10.6% - 15.4%) | 9.4%  (7.3% - 11.8%) | 10.5%  (8.3% - 13.1%) | 22.4%  (19.4% - 25.5%) |
| 2022-06 | 26.6%  (23.4% - 29.5%) | 28.0%  (25.0% - 31.0%) | 12.7%  (10.4% - 15.1%) | 8.8%  (6.8% - 10.8%) | 11.1%  (9.1% - 13.3%) | 13.1%  (10.7% - 15.6%) |
| 2022-07 | 29.7%  (26.1% - 33.3%) | 28.5%  (25.1% - 32.2%) | 14.5%  (12.1% - 17.0%) | 8.7%  (6.6% - 11.0%) | 9.3%  (7.3% - 11.6%) | 8.9%  (6.9% - 11.0%) |
| 2022-08 | 27.7%  (24.3% - 30.8%) | 29.6%  (26.2% - 32.7%) | 13.6%  (11.2% - 15.9%) | 9.2%  (7.1% - 11.2%) | 8.7%  (6.9% - 10.8%) | 11.4%  (9.2% - 13.7%) |
| 2022-09 | 26.1%  (23.0% - 29.5%) | 29.2%  (26.1% - 32.3%) | 13.6%  (11.3% - 16.5%) | 9.7%  (7.6% - 11.9%) | 8.5%  (6.7% - 10.7%) | 13.2%  (11.0% - 15.7%) |
| 2022-10 | 24.2%  (21.3% - 27.4%) | 25.5%  (22.3% - 28.6%) | 13.4%  (11.2% - 15.9%) | 10.0%  (7.9% - 12.3%) | 11.4%  (9.2% - 13.7%) | 15.5%  (12.9% - 18.2%) |
| 2022-11 | 27.3%  (24.0% - 30.6%) | 26.5%  (23.5% - 29.8%) | 11.5%  (9.5% - 13.7%) | 8.0%  (6.0% - 10.0%) | 8.7%  (6.8% - 10.9%) | 17.8%  (15.1% - 20.7%) |
| 2022-12 | 21.3%  (18.3% - 24.8%) | 23.5%  (20.5% - 26.5%) | 11.7%  (9.5% - 14.1%) | 11.4%  (9.3% - 13.9%) | 11.7%  (9.3% - 13.9%) | 20.3%  (17.2% - 23.7%) |

- 1. Modelling outcomes from other studies used to inform the model

An independent whole of population model developed by the Burnet Institute [1, 10, 11] was used to inform multiple elements of the aged care model. The whole of population model has been described in detail elsewhere [1, 10-15], and includes inputs such as vaccine coverage, treatment coverage, test types, testing interventions, isolation compliance and policy changes over time from Victorian data. The model has been calibrated to Victorian diagnoses, hospitalisations, and death data from December 2021 to February 2023 [8]. The model runs over multiple years and reflects the changes in population immunity and variants over time. For this aged care analysis, some components of this previous modelling work were utilized:

- The prevalence of different variants over time was used to determine which variants to simulate incursions of into the aged care model. The variants used in the whole of population model (see Figure 13) reflect the BA.1, BA.2, BA.4/5 and XBF waves. The model also included a fitting process to estimate characteristics of each variant; the infectiousness and immune escape of each variant were determined by sampling over a range of values and maintaining only those consistent with sequencing and wastewater data on the distribution of different variants over time (see Table 4).
- Estimates of community prevalence from the whole of population model were used to (1) model previous infections in aged care and account for exposure-acquired immunity (main body, section **Error! Reference source not found.**); and (2) calibrate to the number of incursions into aged care over time (Appendix B.3).


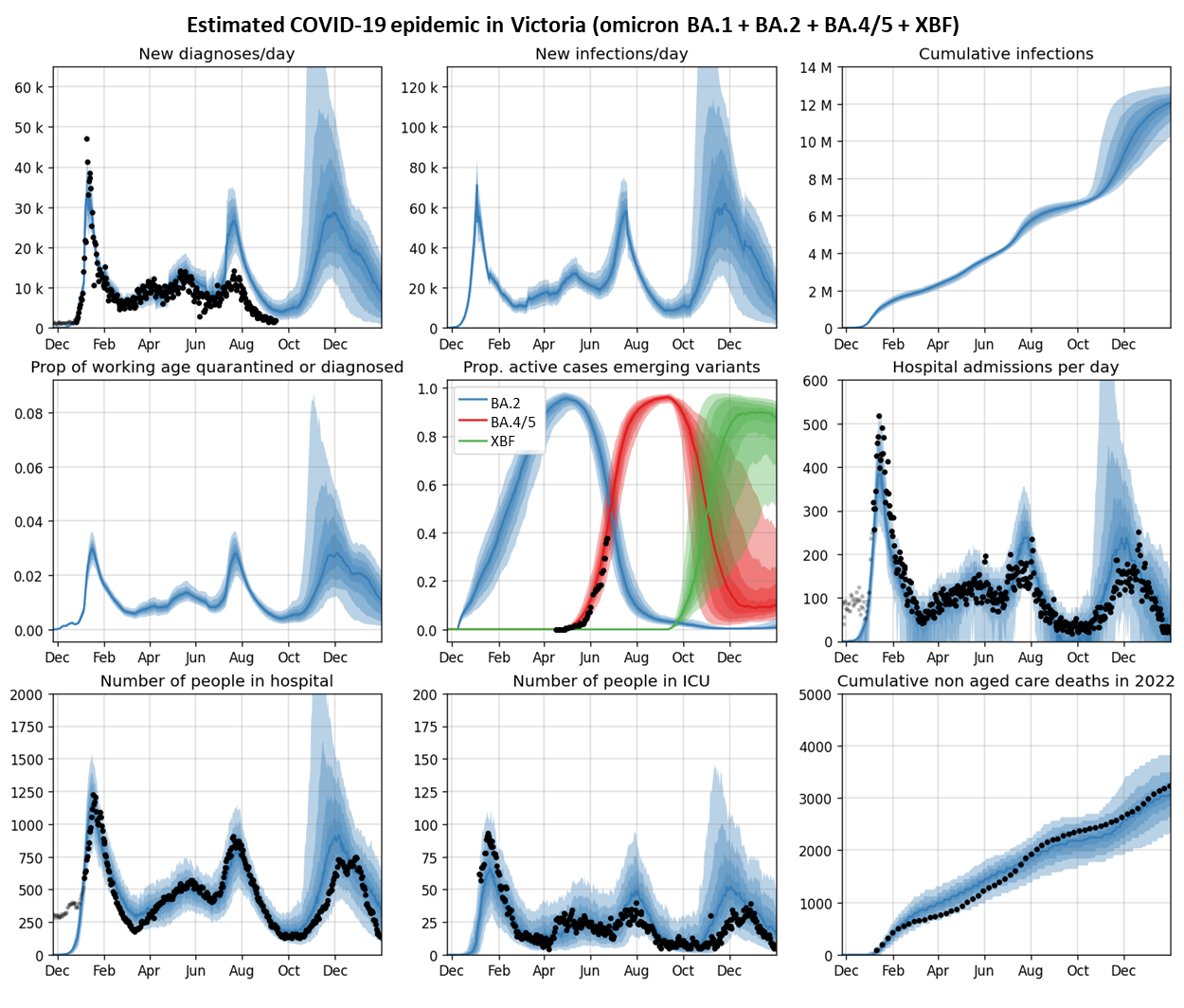


Figure 13: Whole of population model simulated outcomes. Solid lines represent the median with the shading around representing the uncertainty in simulated outcomes. Black scatter points represent Victorian whole of population data provided by Victorian Department of Health [8].

1. Additional results
   1. Additional results: baseline

Table 7: Distribution of infections with uncertainty (aggregate among staff + residents) for incursions occurring in each month between March 2023 and June 2024.

| Number of infections | 1 | 2-3 | 4-5 | 6-7 | 8-9 | 10+ |
| --- | --- | --- | --- | --- | --- | --- |
| 2023-03 | 39.0%  (35.6%, 42.7%) | 20.1%  (17.2%, 22.8%) | 14.0%  (11.6%, 16.4%) | 7.6%  (6.0%, 9.5%) | 5.0%  (3.4%, 6.5%) | 14.1%  (11.8%, 16.6%) |
| 2023-04 | 46.3%  (42.8%, 49.5%) | 20.3%  (17.5%, 23.1%) | 10.4%  (8.4%, 12.4%) | 7.9%  (6.0%, 9.8%) | 5.5%  (4.0%, 7.1%) | 9.6%  (7.6%, 11.6%) |
| 2023-05 | 41.3%  (37.9%, 44.6%) | 21.5%  (18.7%, 24.2%) | 12.0%  (9.8%, 14.3%) | 8.2%  (6.2%, 10.1%) | 4.8%  (3.4%, 6.1%) | 12.3%  (9.9%, 14.6%) |
| 2023-06 | 42.7%  (39.6%, 45.9%) | 21.8%  (18.9%, 24.6%) | 11.5%  (9.3%, 13.6%) | 9.0%  (7.2%, 11.0%) | 6.3%  (4.5%, 7.9%) | 8.6%  (6.9%, 11.0%) |
| 2023-07 | 43.3%  (40.2%, 46.7%) | 21.9%  (19.1%, 24.5%) | 10.9%  (8.8%, 13.0%) | 7.9%  (6.0%, 9.8%) | 5.6%  (4.0%, 7.2%) | 10.4%  (8.3%, 12.5%) |
| 2023-08 | 45.7%  (42.5%, 49.0%) | 21.5%  (18.9%, 24.3%) | 9.4%  (7.5%, 11.4%) | 6.5%  (4.8%, 8.3%) | 5.4%  (3.9%, 7.0%) | 11.7%  (9.6%, 14.0%) |
| 2023-09 | 53.1%  (49.6%, 56.6%) | 24.6%  (21.7%, 27.4%) | 8.9%  (6.9%, 10.9%) | 6.9%  (5.1%, 8.7%) | 4.1%  (2.7%, 5.5%) | 2.3%  (1.4%, 3.3%) |
| 2023-10 | 53.1%  (49.4%, 56.7%) | 25.0%  (21.9%, 28.1%) | 11.8%  (9.6%, 14.1%) | 4.6%  (3.2%, 6.1%) | 2.6%  (1.4%, 3.7%) | 3.0%  (1.9%, 4.2%) |
| 2023-11 | 56.1%  (52.8%, 59.7%) | 23.5%  (20.4%, 26.4%) | 11.8%  (9.4%, 14.1%) | 4.6%  (3.2%, 6.2%) | 2.4%  (1.4%, 3.5%) | 1.7%  (0.8%, 2.6%) |
| 2023-12 | 53.6%  (49.4%, 57.0%) | 25.8%  (22.5%, 29.6%) | 8.4%  (6.6%, 10.3%) | 6.4%  (4.8%, 8.4%) | 3.2%  (2.1%, 4.6%) | 2.5%  (1.4%, 3.7%) |
| 2024-01 | 55.1%  (51.6%, 58.5%) | 22.3%  (19.5%, 25.2%) | 12.0%  (9.6%, 14.3%) | 5.9%  (4.2%, 7.3%) | 2.3%  (1.3%, 3.6%) | 2.5%  (1.5%, 3.6%) |
| 2024-02 | 51.5%  (48.2%, 54.9%) | 23.3%  (20.3%, 26.5%) | 13.2%  (10.6%, 15.5%) | 7.0%  (5.3%, 8.9%) | 2.6%  (1.6%, 3.6%) | 2.6%  (1.5%, 3.9%) |
| 2024-03 | 52.1%  (48.5%, 55.6%) | 26.8%  (23.5%, 30.1%) | 10.5%  (8.4%, 12.7%) | 5.7%  (3.8%, 7.5%) | 2.4%  (1.3%, 3.5%) | 2.5%  (1.4%, 3.9%) |
| 2024-04 | 57.0%  (53.4%, 60.6%) | 23.5%  (20.7%, 26.7%) | 10.0%  (7.8%, 12.1%) | 5.6%  (3.8%, 7.3%) | 3.0%  (1.9%, 4.2%) | 1.1%  (0.4%, 1.9%) |
| 2024-05 | 56.5%  (52.7%, 60.0%) | 24.8%  (21.5%, 27.7%) | 9.5%  (7.4%, 11.9%) | 4.6%  (3.2%, 6.1%) | 2.9%  (1.7%, 4.2%) | 1.9%  (0.9%, 2.8%) |
| 2024-06 | 58.0%  (54.3%, 61.5%) | 22.1%  (19.1%, 25.1%) | 12.2%  (9.9%, 14.5%) | 4.1%  (2.8%, 5.8%) | 2.6%  (1.5%, 3.7%) | 0.9%  (0.3%, 1.6%) |

Table 8: Distribution of diagnoses with uncertainty (aggregate among staff + residents) for incursions occurring in each month between March 2023 and June 2024.

| Number of diagnoses | 0 | 1-2 | 3-4 | 5-6 | 7-9 | 10+ |
| --- | --- | --- | --- | --- | --- | --- |
| 2023-03 | 24.9%  (22.0%, 28.1%) | 29.6%  (26.5%, 32.8%) | 16.3%  (13.7%, 18.8%) | 9.2%  (7.3%, 11.1%) | 8.7%  (7.0%, 10.3%) | 11.3%  (9.1%, 13.5%) |
| 2023-04 | 28.4%  (25.3%, 31.6%) | 29.7%  (26.4%, 32.7%) | 15.1%  (12.5%, 17.5%) | 11.7%  (9.6%, 13.6%) | 8.4%  (6.4%, 10.3%) | 6.4%  (4.6%, 8.3%) |
| 2023-05 | 25.0%  (22.2%, 28.2%) | 33.2%  (30.2%, 36.4%) | 15.8%  (13.3%, 18.3%) | 9.7%  (7.8%, 11.7%) | 9.0%  (7.0%, 11.3%) | 7.4%  (5.6%, 9.3%) |
| 2023-06 | 27.0%  (23.8%, 29.9%) | 33.6%  (30.3%, 37.1%) | 15.4%  (12.8%, 18.0%) | 9.8%  (7.8%, 11.9%) | 9.3%  (7.2%, 11.1%) | 4.9%  (3.5%, 6.5%) |
| 2023-07 | 27.4%  (24.2%, 30.9%) | 32.0%  (29.2%, 34.9%) | 16.0%  (13.5%, 18.6%) | 9.9%  (8.0%, 11.9%) | 8.0%  (6.2%, 10.1%) | 6.6%  (5.0%, 8.3%) |
| 2023-08 | 27.4%  (24.5%, 30.4%) | 32.9%  (29.6%, 36.5%) | 15.4%  (13.2%, 18.1%) | 8.6%  (6.6%, 10.6%) | 7.9%  (5.8%, 9.8%) | 7.9%  (6.1%, 9.7%) |
| 2023-09 | 30.3%  (27.0%, 33.5%) | 38.4%  (34.9%, 42.0%) | 16.4%  (13.9%, 19.0%) | 7.8%  (6.0%, 9.8%) | 5.7%  (4.2%, 7.5%) | 1.3%  (0.6%, 2.1%) |
| 2023-10 | 33.8%  (30.5%, 36.9%) | 39.6%  (36.3%, 42.8%) | 16.0%  (13.5%, 18.5%) | 5.9%  (4.3%, 7.8%) | 3.8%  (2.4%, 5.1%) | 1.0%  (0.3%, 1.8%) |
| 2023-11 | 33.7%  (30.3%, 36.7%) | 40.7%  (37.8%, 44.3%) | 16.7%  (13.8%, 19.1%) | 5.8%  (4.3%, 7.4%) | 2.5%  (1.4%, 3.8%) | 0.7%  (0.1%, 1.4%) |
| 2023-12 | 32.2%  (28.6%, 35.5%) | 43.5%  (39.7%, 47.3%) | 13.7%  (11.5%, 16.2%) | 6.0%  (4.4%, 7.6%) | 3.8%  (2.5%, 5.2%) | 1.0%  (0.4%, 1.8%) |
| 2024-01 | 33.4%  (30.2%, 36.4%) | 42.3%  (38.9%, 45.7%) | 13.6%  (11.3%, 15.9%) | 6.3%  (4.8%, 8.0%) | 3.5%  (2.3%, 4.8%) | 0.9%  (0.3%, 1.7%) |
| 2024-02 | 32.8%  (29.7%, 36.3%) | 40.1%  (36.1%, 44.1%) | 15.1%  (12.7%, 17.5%) | 7.7%  (5.8%, 9.6%) | 3.2%  (1.9%, 4.4%) | 0.9%  (0.4%, 1.7%) |
| 2024-03 | 32.3%  (28.6%, 35.9%) | 42.6%  (39.0%, 46.0%) | 14.9%  (12.4%, 17.9%) | 6.5%  (4.9%, 8.3%) | 2.9%  (1.8%, 4.3%) | 0.7%  (0.1%, 1.3%) |
| 2024-04 | 34.1%  (30.9%, 37.8%) | 41.3%  (38.1%, 45.1%) | 15.2%  (12.7%, 17.8%) | 6.4%  (4.5%, 8.4%) | 2.6%  (1.6%, 3.8%) | 0.3%  (0.0%, 0.7%) |
| 2024-05 | 34.3%  (30.4%, 37.9%) | 44.0%  (40.5%, 47.8%) | 12.8%  (10.6%, 15.2%) | 5.8%  (4.2%, 7.7%) | 2.7%  (1.6%, 3.9%) | 0.4%  (0.1%, 0.9%) |
| 2024-06 | 33.5%  (30.0%, 37.4%) | 43.7%  (39.9%, 47.6%) | 14.4%  (12.1%, 17.2%) | 5.6%  (3.8%, 7.5%) | 2.4%  (1.3%, 3.5%) | 0.5%  (0.0%, 1.0%) |

- 1. Additional results: scenarios


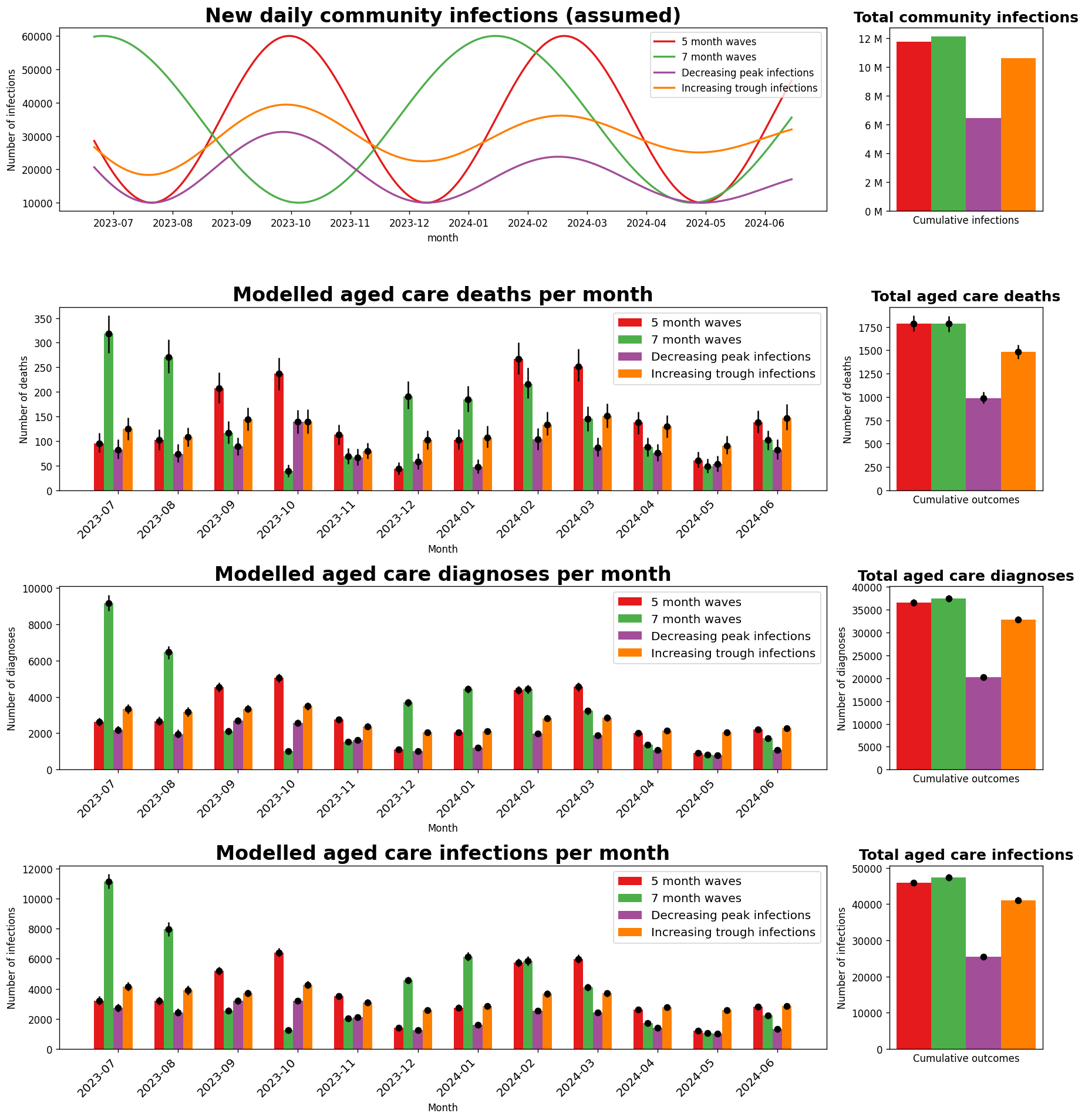


Figure 14: Community prevalence scenarios. Deaths, diagnoses, and infections within RACFs follow similar trends as community prevalence waves.


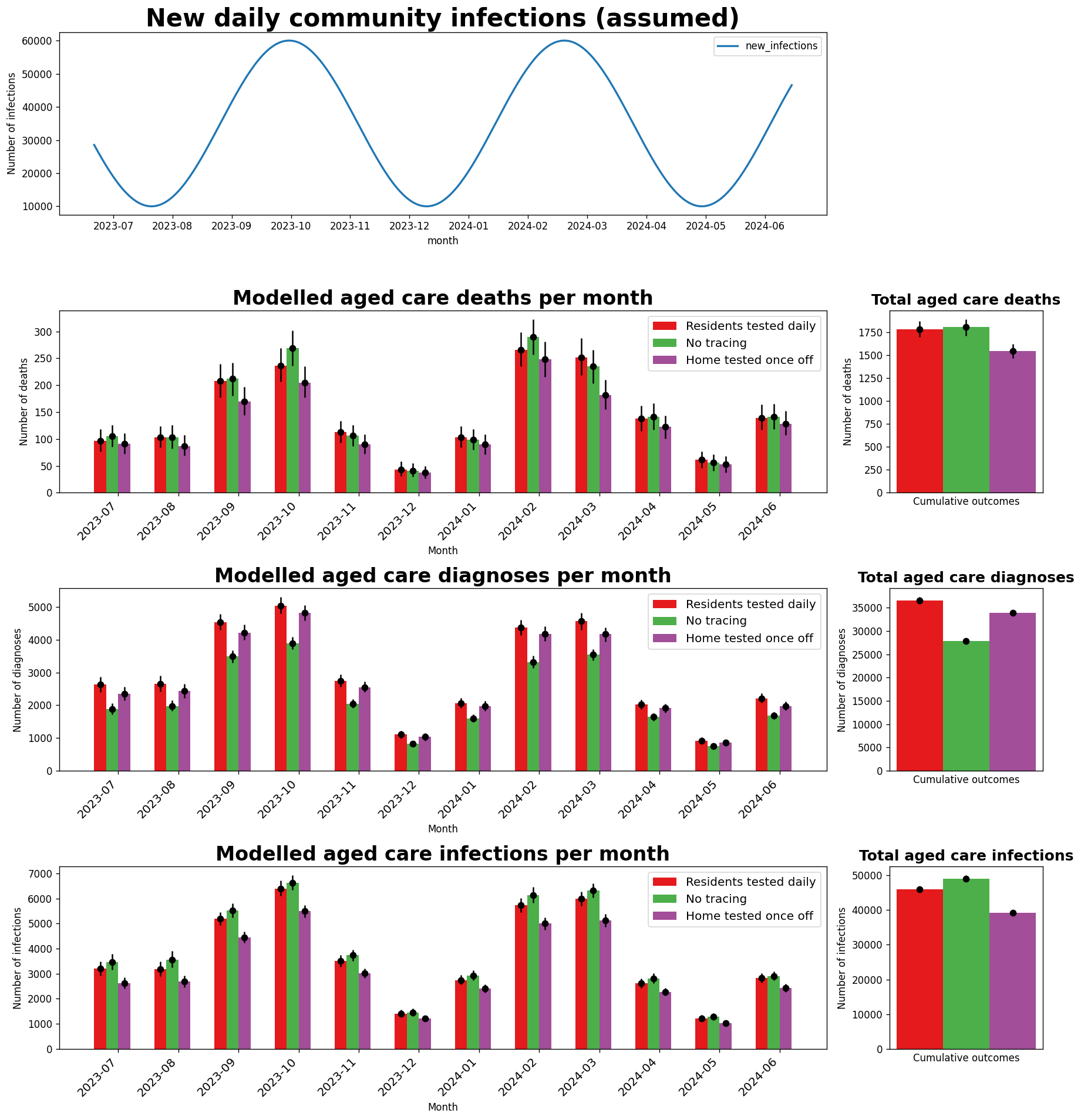


Figure 15: Contact tracing scenarios. No tracing exhibits a smaller number of diagnoses per month, with the greatest number of deaths and infections. Testing the home once has a greater impact in than testing residents daily, with the greatest impact seen in mid-2023.


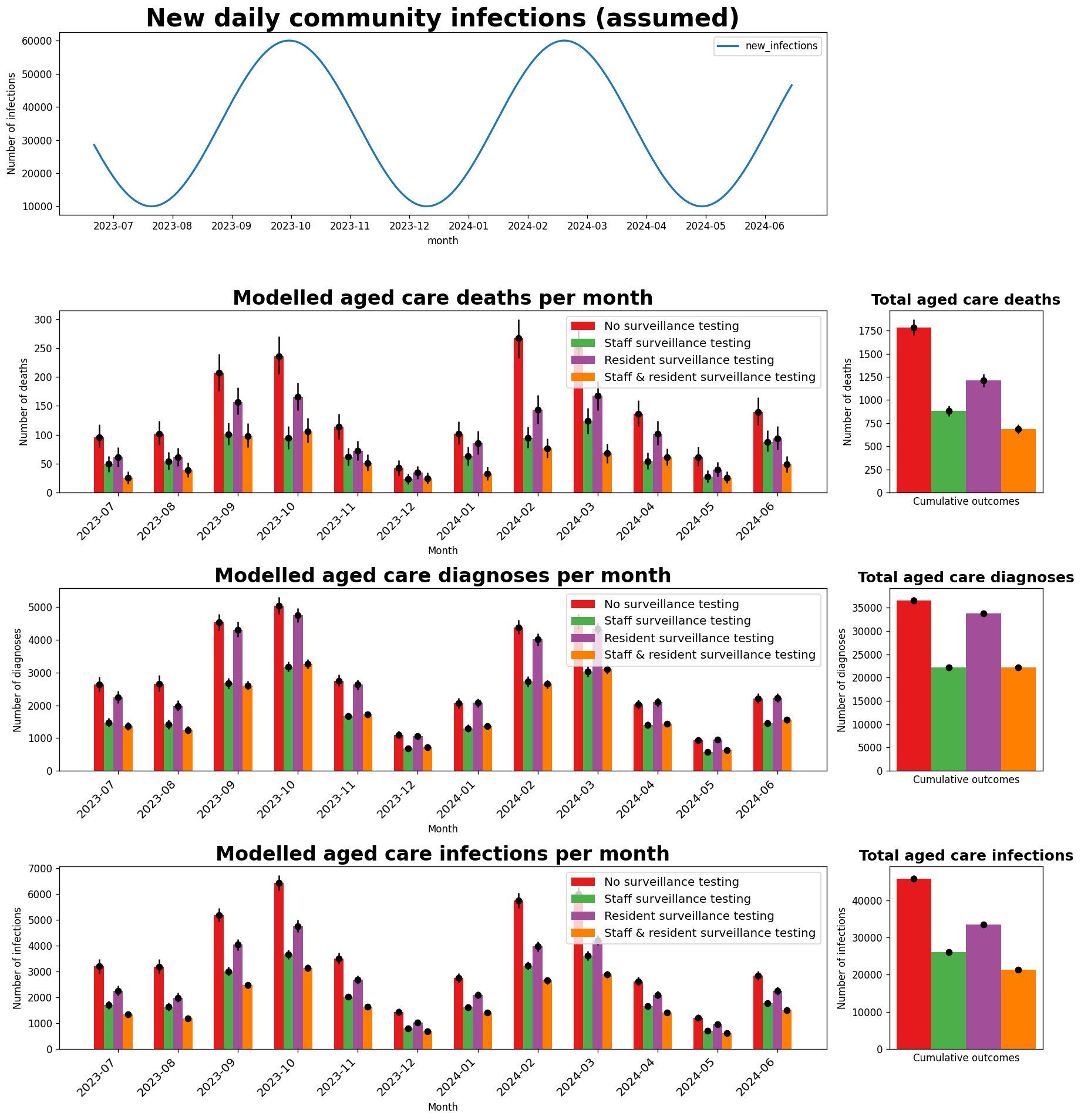


Figure 16: Surveillance testing scenarios. Surveillance testing shows various levels of impact, with no surveillance testing having the greatest number of deaths, diagnoses, and infections in every month. Testing staff and residents shows the greatest improvements in deaths and infections.


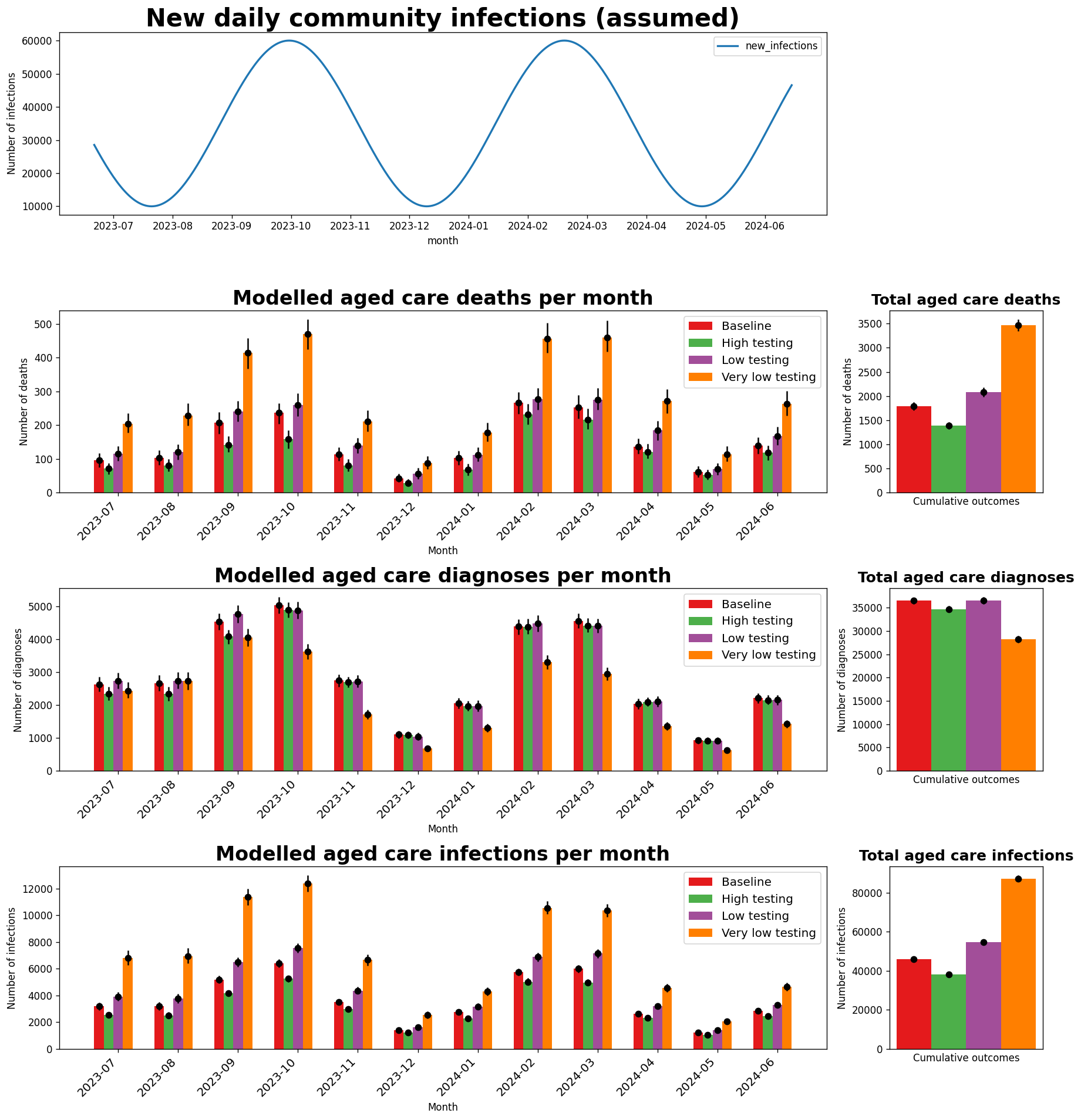


Figure 17: Symptomatic testing scenarios. Small differences are seen each month between the baseline, high testing, and low testing scenarios, with high testing having the best outcomes. Very low testing has a negative impact on outcomes in RACFs, with the largest number of deaths and infections of the four scenarios each month.


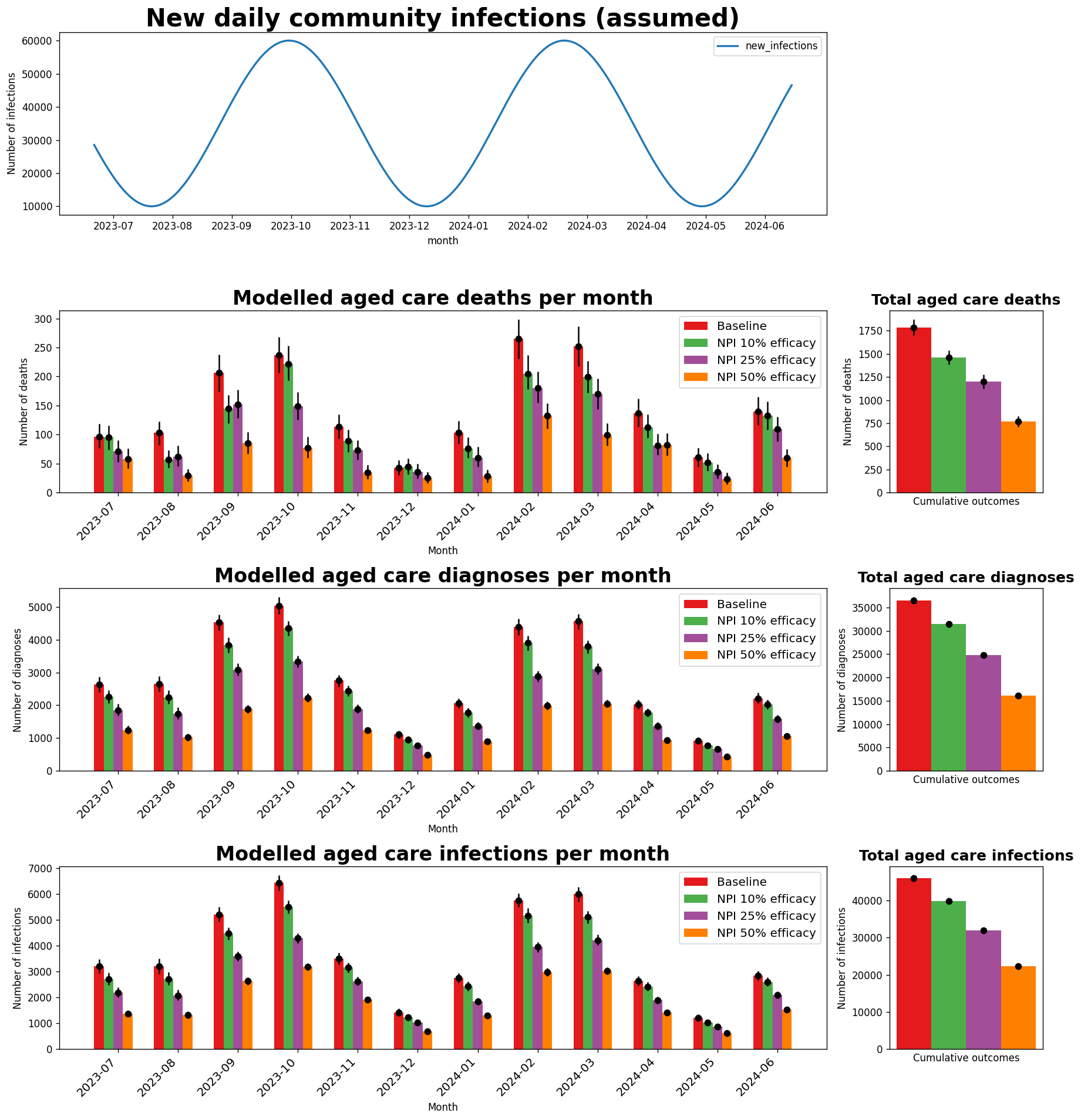


Figure 18: NPI efficacy scenarios. NPIs of various efficacies reduced deaths, diagnoses, and infections linearly. NPIs with 50% efficacy showed the greatest efficacy with outcomes half that of the baseline each month after the introduction.


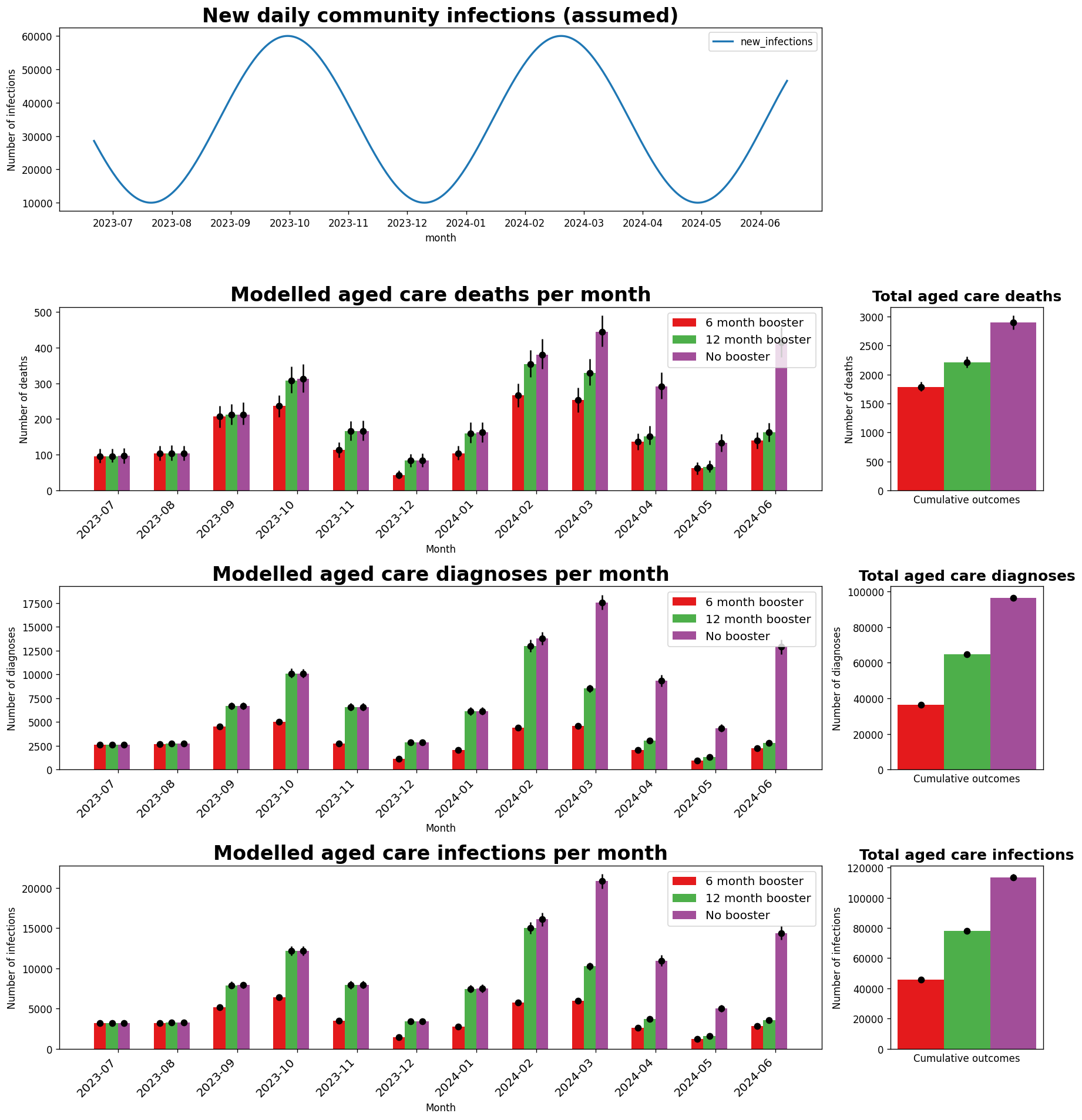


Figure 19: Vaccine booster scenarios. Boosters every 6-months shows the greatest reduction in adverse outcomes compared to the other scenarios. Once the 12-month booster is introduced in February 2023 the outcomes approach that of the 6-month booster from the immunity boost. No boosters result in a large increase in adverse outcomes from the residents gaining no additional immunity boosts other than previous infections.

- 1. Sensitivity analyses figures


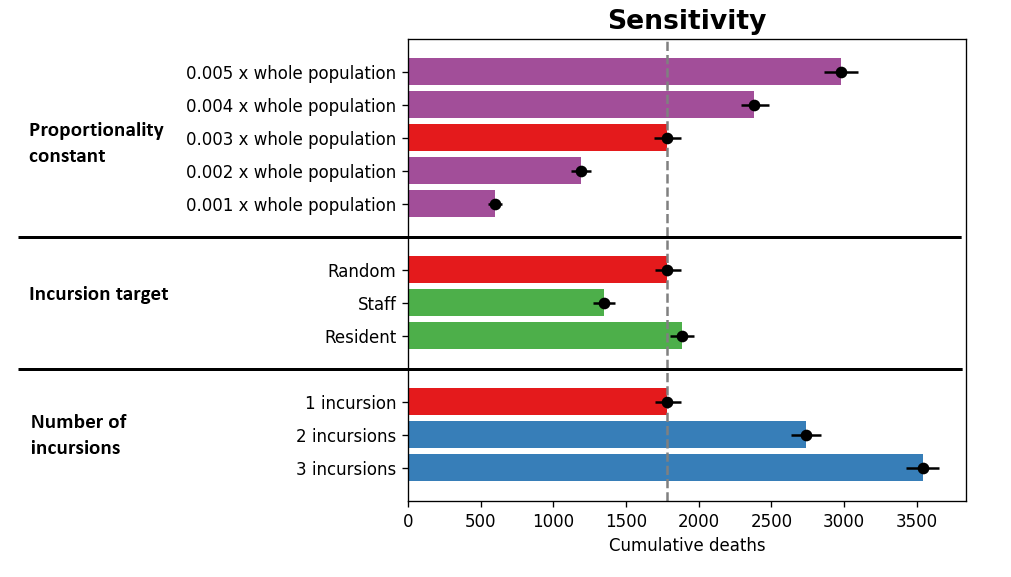


Figure 20: Sensitivity analyses bar graphs. Combined outcomes between July 2023 to June 2024 for sensitivity analyses. The red bar represents the outcomes from baseline assumptions and each additional bar represents outcome from a specific sensitivity.

Table 9: Sensitivity analyses outcomes compared to baseline. Percentage change in deaths, diagnoses and infections compared to the baseline.

| **Scenario** | | **Difference compared to baseline scenario** | | |
| --- | --- | --- | --- | --- |
|  |  | **Infections** | **Diagnoses** | **Deaths** |
| **Incursion constant** | 0.005 x whole population | 66%  (64%, 69%) | 66%  (64%, 69%) | 66%  (60%, 72%) |
|  | 0.004 x whole population | 33%  (31%, 35%) | 33%  (31%, 35%) | 33%  (27%, 39%) |
|  | 0.003 x whole population | Baseline | Baseline | Baseline |
|  | 0.002 x whole population | -33%  (-34%, -31%) | -33%  (-34%, -31%) | -33%  (-37%, -29%) |
|  | 0.001 x whole population | -66%  (-67%, -65%) | -66%  (-67%, -65%) | -66%  (-69%, -63%) |
| **Incursion target** | Random | Baseline | Baseline | Baseline |
|  | Staff | -27%  (-29%, -25%) | -24%  (-26%, -22%) | -24%  (-28%, -19%) |
|  | Resident | 39%  (37%, 41%) | 33%  (31%, 35%) | 5%  (0%, 9%) |
| **Number of incursions** | 1 incursion | Baseline | Baseline | Baseline |
|  | 2 incursions | 64%  (61%, 66%) | 65%  (63%, 68%) | 53%  (47%, 59%) |
|  | 3 incursions | 115%  (113%, 118%) | 118%  (116%, 121%) | 98%  (91%, 104%) |


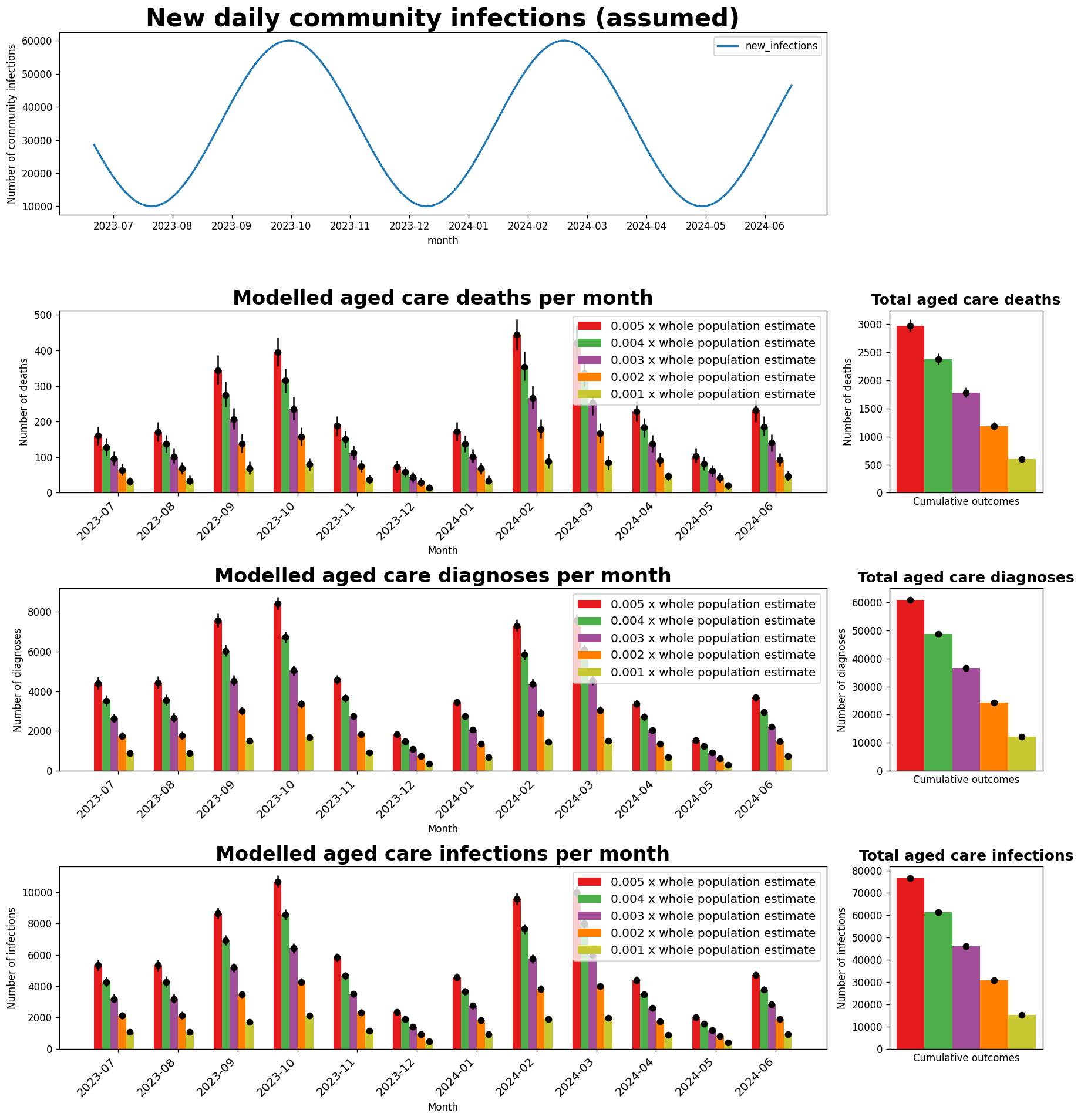


Figure 21: Incursion constant scenarios. Number of deaths, diagnoses and infections decrease linearly with decrease in incursion constant.


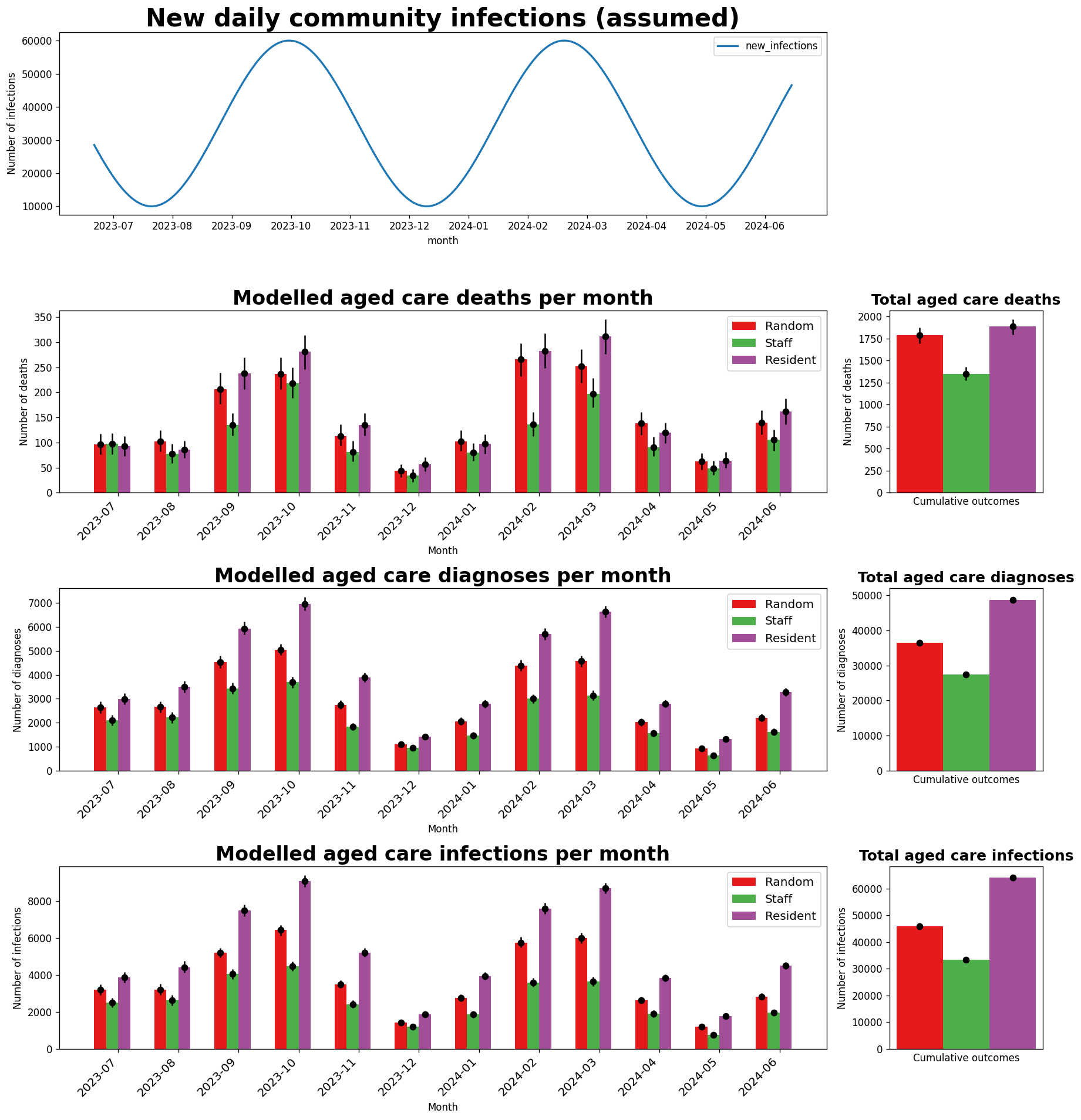


Figure 22: Incursion target scenarios. Model is sensitive to the incursion target, with deaths, diagnoses and infections increasing when the first infection is always a resident and outcomes decreasing when initial infection is a staff member.


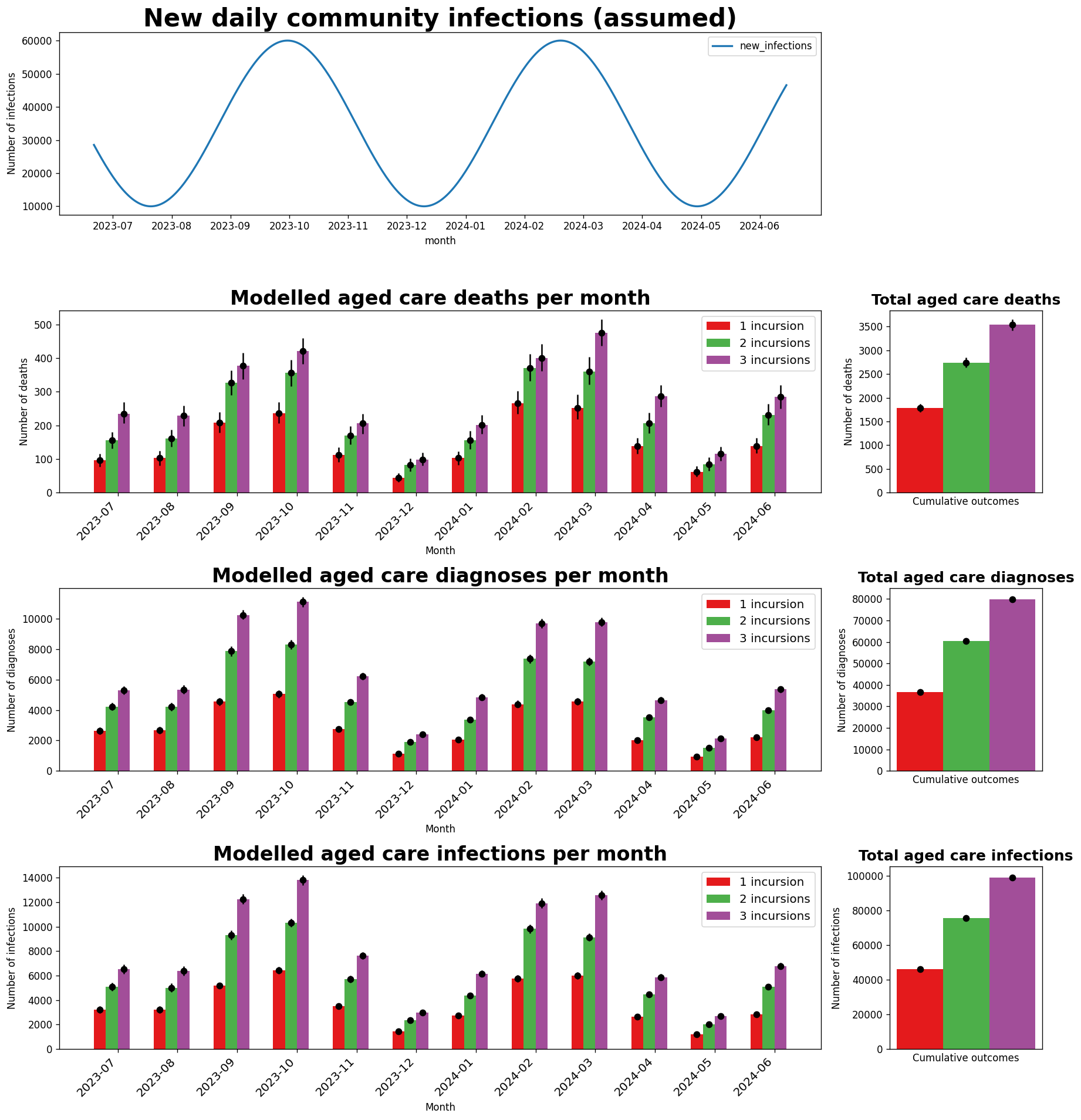


Figure 23: Number of incursions scenarios. Deaths, diagnoses and infections increase linearly with increased number of initial infections.

# Supplement references

1. Scott, N., et al., *COVID-19 epidemic modelling for policy decision support in Victoria, Australia 2020-2021.* BMC Public Health, 2023. **23**(1): p. 988.

2. Arevalo-Rodriguez, I., et al., *False-negative results of initial RT-PCR assays for COVID-19: A systematic review.* PLoS One, 2020. **15**(12): p. e0242958.

3. Muhi, S., et al., *Multi-site assessment of rapid, point-of-care antigen testing for the diagnosis of SARS-CoV-2 infection in a low-prevalence setting: A validation and implementation study.* Lancet Reg Health West Pac, 2021. **9**: p. 100115.

4. Cohen, J.A., et al., *Quantifying the role of naturally-and vaccine-derived neutralizing antibodies as a correlate of protection against COVID-19 variants.* 2021.

5. UK SAGE, *Public Health England estimates for duration of protection of covid-19 vaccines against clinical disease*. 2021.

6. Bar-On, Y.M., et al., *Protection of BNT162b2 Vaccine Booster against Covid-19 in Israel.* N Engl J Med, 2021. **385**(15): p. 1393-1400.

7. Barda, N., et al., *Effectiveness of a third dose of the BNT162b2 mRNA COVID-19 vaccine for preventing severe outcomes in Israel: an observational study.* Lancet, 2021. **398**(10316): p. 2093-2100.

8. Department of Health, *Personal Communication with Department of Health*, B. Institute, Editor. 2022.

9. Wong, C.K.H., et al., *Real-world effectiveness of molnupiravir and nirmatrelvir plus ritonavir against mortality, hospitalisation, and in-hospital outcomes among community-dwelling, ambulatory patients with confirmed SARS-CoV-2 infection during the omicron wave in Hong Kong: an observational study.* Lancet, 2022. **400**(10359): p. 1213-1222.

10. Scott, N., et al., *Modelling the impact of relaxing COVID-19 control measures during a period of low viral transmission.* Med J Aust, 2021. **214**(2): p. 79-83.

11. Abeysuriya, R.G., et al., *Preventing a cluster from becoming a new wave in settings with zero community COVID-19 cases.* BMC Infect Dis, 2022. **22**(1): p. 232.

12. Abeysuriya, R.G., et al., *Keeping kids in school: modelling school-based testing and quarantine strategies during the COVID-19 pandemic in Australia.* Front Public Health, 2023. **11**: p. 1150810.

13. Delport, D., et al., *Lives saved by public health restrictions over the Victorian COVID-19 Delta variant epidemic wave, Aug-Nov 2021.* Epidemics, 2023. **44**: p. 100702.

14. Kerr, C.C., et al., *Covasim: An agent-based model of COVID-19 dynamics and interventions.* PLoS Comput Biol, 2021. **17**(7): p. e1009149.

15. Institute for Disease Modelling. *Covasim model*. Available from: <https://github.com/InstituteforDiseaseModeling/covasim>.

1. Nonlinear functions translate NAb levels into immunity against infection, symptoms, and disease severity. A 95% reduction in NAbs does not correspond to a vaccine only being 5% effective against a variant. [↑](#footnote-ref-2)
